# *Dichondra repens* J.R.Forst. and G.Forst.: A Review of Its Traditional Uses, Chemistry, Pharmacology, Toxicology and Applications

**DOI:** 10.3389/fphar.2020.608199

**Published:** 2021-02-08

**Authors:** Qi Yao, Ying Wang, Zhiyu Dong, Chencen Lai, Botao Chang, Qiuju Gong, Shuaijun Ren, Dongxue Sun, Jie Lu, Ying Gao

**Affiliations:** ^1^ The First Affiliated Hospital, Guizhou University of Traditional Chinese Medicine, Guiyang, China; ^2^ Department of Gynaecology and Obstetrics, Taizhou Traditional Chinese Medicine Hospital, Taizhou, China; ^3^ Department of Pharmacy, Guizhou University of Traditional Chinese Medicine, Guiyang, China

**Keywords:** *Dichondra repens* forst, chemistry, pharmacology, toxicity, Applications

## Abstract

**Ethnopharmacology relevance:** *Dichondra repens* J.R.Forst. and G.Forst (DRF; Convolvulaceae, called Matijin in Chinese), has been traditionally used to treat jaundice, bacillary dysentery, urinary tract infection, edema, contusions, and strains and sprains based on traditional Chinese medicine (TCM) concepts.

**Aim of study:** This paper intends to provide a comprehensive and critical analysis of research on DRF focusing on a relationship between traditional uses and pharmacological effects, evaluating the therapeutic potential of this plant.

**Methods:** Relevant data on DRF were retrieved from available databases.

**Results:** The heat-clearing and detoxifying, and removing the phlegm and turbid urine effects of DRF are linked to its anti-hepatitis B virus (HBV), anti-inflammatory, and hepatoprotective activities. Especially, the hepatoprotective effects of DRF are mainly based on anti-HBV activities of phenylalanine dipeptides Matijin-Su (MTS) and its derivatives derived from this plant. Further, a phase I anti-HBV clinical trial of a candidate compound named bentysrepinine (Y101, Chinese name Tifentai) has been completed. Also, anti-tumor, analgesic, and antibacterial properties have been reported in the extracts and compounds from DRF. Although pharmacy, pharmacodynamics, toxicology, and pharmacokinetics of bentysrepinine have been systemically reported, no studies have reported chemistry, safety, pharmacology of other compounds or extracts systemically.

**Conclusion:** Phenylalanine dipeptide compounds are main components and MTS is a characteristic substance of DRF. The main pharmacological effect of DRF is anti-HBV activity, which is coherent with the traditional use of this plant in China. Except bentysrepinine, few studies have been conducted on toxicities of the extracts or compounds from DRF. Thus, it is still necessary to evaluate safety, chemistry, pharmacology of the extracts or compounds from DRF regarding the link between traditional uses and modern applications before the future clinical trials. Bacterial sepsis, cholecystitis and tumors may be prior therapeutic targets of this plant in the future.

## Introduction

The genus *Dichondra* contains fourteen species: most in North and South America, two species in New Zealand, one in Australia, and one in China. It has been confirmed that specimens from China were previously called *Dichondra repens* as D. micrantha Urban (Tharp and Johnston, 1961). Genuine D. *repens* J. R. and G. Forster is confined to Australia and New Zealand according to their taxonomic concepts.

*Dichondra repens* J.R. Forst. and G. Forst (DRF) is a perennial creeping herb (The Plant List, 2012). Its common names include hebaocao or jinsuoshi, huangdancao, rouhundun, xiaojinqiancao distinct from jinqiancao (Lysimachia christinae Hance). Conventionally, its whole plant is used. TCM characteristics of DRF is summarized as bitter and spicy in flavor, slightly cold in nature, and attributed to lung and stomach meridians (Editorial board of Chinese materia medica and State administration of traditional Chinese medicine, 2005). It has the functions of heat-clearing and detoxifying, removing the phlegm and turbid urine, reducing fever and causing diuresis, and promoting tissue regeneration and hemostasis (Fujian institute of medicine, 1970; Guangxi health administration, 1970; The minister of health of logistics department of Kunming military command, 1970). Nowadays, it is used together with other TCMs to cure HBV infection-related diseases in clinic (Cheng et al., 1996). In subtropical and mediterranean regions, DRF is used as a potted plant for house decoration for its prostrate growth habit and no need for mowing (Cardin et al., 2005).

At present, pharmacological studies have shown that compounds or extracts from DRF exhibit anti-viral (Qiu et al., 2016), anti-bacterial (Qu et al., 2003a; Wu et al., 2009), anti-inflammatory (Sheu et al., 2012), analgesic (Qu et al., 2003a; Zeng et al., 2005), antipyretic (Qu et al., 2003b), antioxidant (Qu et al., 2003a; Sheu et al., 2012), anti-tumor (Qiu et al., 2016; Xu et al., 2014), hepatoprotective (Qu et al., 2003c, Qu et al., 2003d; Zeng et al., 2011), cholagogic (Qu et al., 2003b), and immnomodulatory effects (Qu et al., 2003b). However, most of these pharmacological studies are still remained at cell or animal level, there is a huge gap for its application in treating diseases in clinic. Thus, more detailed information on pharmacological evaluations of this plant are needed to clarify the significance of this plant in treating various diseases in clinic.

In this paper, we manage to provide a critical analysis of DRF, including traditional uses, botany, chemistry, pharmacology, modern applications, limitations of studies, and future research directions by using available information from retrieved literatures regarding traditional usage and modern research. Furthermore, the relationship between the traditional uses and the modern applications of DRF is also focused. In view of this, we expect to provide some evidences for therapeutic potential of this plant as a new drug in the future.

## Traditional Uses

Commonly, DRF is distributed in the south of the Yangtze River in China, especially in Yunnan, Guizhou, Guangxi, Sichuan, Fujian, Zhejiang, and Hunan provinces. It often grows in areas between 1,300 and 1980 m above the sea level, mostly on hillsides, grasslands or furrows (Flora of China Editorial Committee, The Chinese Academy of Sciences, 1997). Now, it has been artificially cultivated for drought tolerance (Li et al., 2007).

DRF is respectively called “Panuo” and “Wobisheliu” in traditional medicines of Dai and Miao nationalities (Editorial board of Chinese materia medica and State administration of traditional Chinese medicine, 2005; Qiu and Du, 2005), two ethnic groups in Southwestern China. It was first reported in a herbal medicine work named “A supplement to the compendium of materia medica” in Qing Dynasty and rearranged in 1998.

In concepts of Dai and Miao medicines, DRF is traditionally used to cure “Longniu” (urinary tract infections and urinary calculi), “Longmengshahei” (abdominalgia, diarrhea, and bacillary dysentery), “Longandale” (jaundice), “Longhaimaimaoba” (high fever), “Longhaixian” (malaria), “Longshalongjiehuo” (sore throat), “Longshalongjiehougaiban, ole” (swollen gums, bleeding), “Longshalongdajiebangliang” (swelling and pain of eye), “Shuofenglinglan” (canker sore), and “Nalemaoshamotalongtafeixiang” (wind-fire superabundant-type menstrual disorder) (Editorial board of Chinese materia medica and State administration of traditional Chinese medicine, 2005; Qiu and Du, 2005; Yu, 2016) (Table 1). In concept of traditional medicine of Han nation, it is used for treating dysentery, jaundice, furuncle in eye or finger, edema, hematuria, abdominal distension, and other diseases (Table 2) (Guangxi health administration, 1970; The minister of health of logistics department of Kunming military command, 1970; Fujian institute of medicine, 1970). Therefore, current status of folk medicinal usage of this plant in China is consistent with its traditional uses.

**TABLE 1:** The traditional uses of DRF in Dai and Miao nations in China.

No	Composition(s)	Traditional uses	Usage(s)	References(s)
1	DRF 15g, *Siegesbeckia orientalis* L. 10 g, *Plantago asiatica* L. 15 g, *Verbena officinalis* L. 15 g	Curing “Longniu” (urinary tract infections and urinary calculi) and “Longmengshahei” (abdominalgia, diarrhea, and bacillary dysentery)	Decoction and take orally	Editorial board of Chinese materia medica and state administration of traditional Chinese medicine (2005)
2	DRF 30 g, *Tadehagi triquetrum* (L.) H.Ohashi 15 g, *Senna tora* (L.) Roxb. 15 g, *Imperata cylindrica* (L.) Beauv. 15 g, *Isatis tinctoria* L. 20 g	Curing “Longandale” (jaundice)	Decoction and take orally	Editorial board of Chinese materia medica and state administration of traditional Chinese medicine (2005)
3	DRF 20g, *Lagenaria siceraria* (Molina) Standl. 15 g, *Saccharum officinarum* L. 20 g, *Acorus calamus* var. angustatus Besser 10 g	Curing “Longhaimaimaoba” (high fever)	Decoction and take orally	Editorial board of Chinese materia medica and state administration of traditional Chinese medicine (2005)
4	DRF 30 g	Curing “Longhaixian” (malaria)	Decoction and take orally with a small amount of salt	Editorial board of Chinese materia medica and state administration of traditional Chinese medicine (2005)
5	DRF 15 g	Curing “Longshalongjiehuo” (sore throat), “Longshalongjiehougaiban, ole” (swollen gums, bleeding), “Longshalongdajiebangliang” (swelling and pain of eye), “shuofenglinglan” (canker sore)	Mashing and take orally with boiled water	Editorial board of Chinese materia medica and state administration of traditional Chinese medicine (2005)
6	DRF 50 g, *Hypericum japonicum* Thunb. 50 g, *Plantago asiatica* L. 25 g, *Imperata cylindrica* (L.) P.Beauv. 20 g	Curing acute icteric hepatitis	Decoction and take orally	Qiu and Du (2005)
7	DRF 15g, *Lysimachia christinae* Hance 30g, *Plantago asiatica* L. 25g, *Imperata cylindrica* (L.) P.Beauv. 20 g	Curing urinary calculi	Decoction and take orally	Qiu and Du (2005)
8	DRF 15 g, *Plantago asiatica* L. 15 g, *Cirsium japonicum* DC. 50 g, P*yrrosia lingua* (Thunb.) Farw. 15 g, lean pork 200 g	Curing chronic glomerulonephritis	Stewing and take soup orally at morning and night	Qiu and Du (2005)
9	DRF 50 g, *Hydrocotyle sibthorpioides* Lam. 50 g, lean pork 200 g	Curing acute hepatitis B	Stewing and take soup and meat orally	Qiu and Du (2005)
10	(i) A certain amount of fresh DRF. (ii) DRF 1550 g	Curing nephritic edema	(i) Mashing and apply on the belly button for 7 days and once a day. (ii) Decoction and take orally	Qiu and Du (2005)
11	DRF 1030 g, *Gypsophila paniculata* L. 1030 g, *Centella asiatica* (L.) Urb. 1030 g, *Taraxacum mongolicum* Hand.-Mazz. 1030 g	Curing biliary calculus and vesical calculus	Decoction and take orally	Qiu and Du (2005)
12	DRF 10 g, *Schisandra propinqua* subsp. Sinensis (Oliv.) R.M.K.Saunders 10 g, *Mezoneuron cucullatum* (Roxb.) Wight and Arn. 10 g	Curing lumps	Sparkling wine and take orally	Qiu and Du (2005)
13	DRF 30 g, *Citrus aurantium* L. 15 g, *Hemerocallis fulva* (L.) L. 15 g	Curing jaundice	Decoction and take orally	Qiu and Du (2005)
14	DRF 30 g	Curing menstrual disorder	Sparkling wine and take orally	Qiu and Du (2005)
15	DRF 20 g, *Eclipta prostrata* (L.) L. 15 g, *Aster indicus* L. 15 g, *Oxalis corniculata* L. 15 g, *Leonurus japonicus* Houtt. 20 g	Curing “Nalemaoshamotalongtafeixiang” (wind-fire superabundant-type menstrual disorder)	Decoction and take orally every 8 h for three times	Yu (2016)

The full taxonomic names of the species have been validated using www.theplantlist.org.

**TABLE 2:** The traditional uses of DRF in Han nation in China.

No	Composition(s)	Traditional use(s)	Usage(s)	References(s)
1	DRF 50～100 g	Curing icteric hepatitis, chronic cholecystitis, bacillary dysentery, Contusions and strains, traumatic bleeding	Decoction and take orally or apply powder on the wound when traumatic bleeding	The minister of health of logistics Department of Kunming military Command (1970)
2	25～50 g DRF	Curing acute icteric hepatitis, acute cholecystitis, urinary tract calculi, pneumorrhagia	Decoction and take orally	Guangxi health administration (1970)
3	(i) DRF 50～100 g. (ii) DRF 50～100 g, Erigeron annuus (L.) Pers. 50 g, Lygodium japonicum (Thunb.) sw. 50 g, brown sugar 25 g	Curing “Yanghuang” (hot- and damp- type jaundice)	(i) Decoction and take orally. (ii) Decoction and take orally	Fujian institute of medicine (1970)
4	DRF 50～100 g	Curing common cold with wind-heat syndrome	Decoction and take orally	Fujian institute of medicine (1970)
5	DRF 100～150 g	Curing dysentery	Mashing and extracting juice; take orally with rock sugar and boiled water	Fujian institute of medicine (1970)
6	DRF 100 g	Curing abdominal pain caused by heat stroke	Mashing and extracting juice; take orally with wine and boiled water	Fujian institute of medicine (1970)
7	DRF 50～100 g, rock sugar 25 g	Curing hematuria	Decoction and take orally	Fujian institute of medicine (1970)
8	DRF 100～150 g	Curing sore throat	Mashing and extracting juice; take orally with honey	Fujian institute of medicine (1970)
9	DRF 50～100 g	Curing “Ruyong” (acute suppurative mastitis)	Decoction with wine moderately and take orally after meals	Fujian institute of medicine (1970)
10	DRF 50～100 g	Curing stomatitis	Mashing and extracting juice; rinse the mouth frequently	Fujian institute of medicine (1970)
11	DRF 50～100 g	Curing acute otitis media	Mashing and extracting juice; Wash external auditory meatus and drip in ear	Fujian institute of medicine (1970)

The full taxonomic names of the species have been validated using www.theplantlist.org.

Nowadays, DRF is used to treat acute icteric hepatitis, acute cholecystitis, urolithiasis, pneumorrhagia (whole plant in decoction in Guangxi) (Guangxi health administration, 1970), bacillary dysentery and traumatic injury (whole plant in decoction in Yunnan) (The minister of health of logistics department of Kunming military command, 1970), wind-heat type common cold, heat stroke-induced abdominalgia, hematuria, sore throat, stomatitis, acute otitis media (whole plant in decoction in Fujian) (Fujian institute of medicine, 1970), analgesia, anti-inflammation and bacteriostasis (n-butanol extracts from whole plant; petrol ether extract from whole plant; ethanol extracts of from whole plant) (Qu et al., 2003a; Zeng et al., 2005; Sheu et al., 2012), hepatic injury (n-butanol extracts from whole plant) (Qu et al., 2003c; Qu et al., 2003d). Usually, the whole plant of DRF is used partly due to lacks in studies on active components in different parts of this plant.

## Botany

**FIGURE 1:**
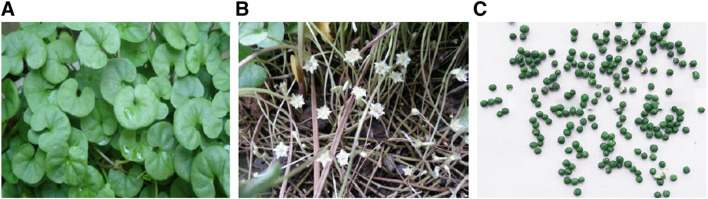
Images of DRF **(A)** whole-plant of DRF; **(B)** flowers of DRF; **(C)** fruits of DRF.

DRF is a perennial creeping herb. Its slender stems are covered by gray pubescent. Adventitious roots occur when nodes grow on the ground. Leaves are round or kidney-shaped, 4～25 mm in diameter, broadly rounded or emarginate at apex and broadly heart-shaped at base. The surface of the leaf is slightly glabrous and its back is pubescent; Petioles are usually (1.5) 3～5 (6) cm long (Figure 1A).

Solitary flowers are in axils of leaves, filiform stalks are shorter than petioles; Obtuse sepals (2～3 mm long) are obovate to oblong to spoon-shaped, abaxially and marginally pubescent; Campanulate corolla is yellow and quinquepartite and slightly longer than calyx. Its lobes are oblong-lanceolate and glabrous. Five stamens grow at bend of lobes of corolla 2. Its filaments are short and equal in length; Ovary is sparsely pilose and 2-loculed (2 ovules/locule) (Figure 1B). Membranous capsule (fruit) which is appropriately 1.5 mm in diameter is nearly spherical, small, and shorter than calyx (Figure 1C). The flowering and fruiting seasons of DRF are from April to May and from July to August, respectively. At present, no report has been documented on chemical components as well as pharmacological activities of flowers or fruits of DRF.

## Chemistry

**FIGURE 2:**
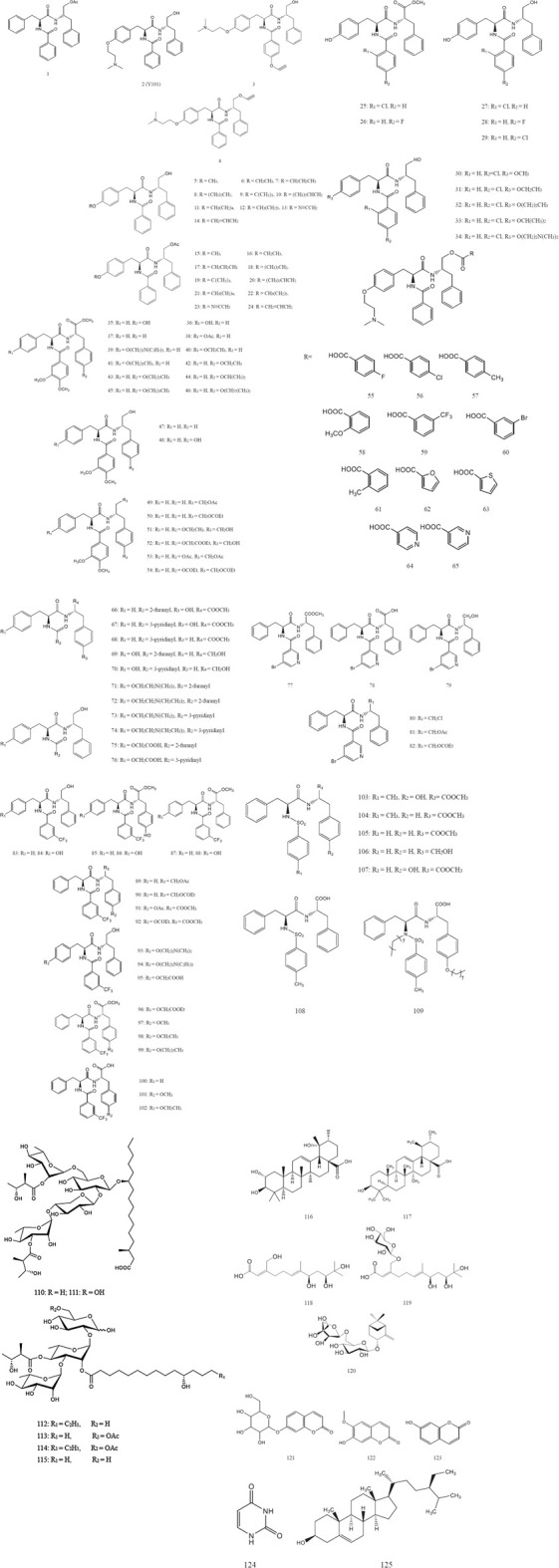
The isolated and synthesized compounds derived from DRF.

Now, based on the traditional uses chemical components and pharmacological effects of DRF have been investigated. Chemistry studies on DRF have isolated and synthesized 125 phenylalanine dipeptide compounds including MTS and its derivatives (1～109), six resin glycosides (110～115), five terpenoids (116～120), three coumarins (121～123), one uracil compound (124), and one steroid (125). All of the separated compounds were isolated from the whole plant. Nowadays, flowers and fruits of DRF have not been studied individually. Thus, it is necessary to strengthen chemical studies on the components from the flowers and fruits of this plant. The chemical structures and names of these compounds (1～125) are listed as follows (Figure 2 and Table 3). In addition, 62 volatile components were separated from DRF (Table 4, 5).

**TABLE 3:** The isolated and synthesized compounds derived from DRF.

No	Name	Parts of plant	Source	References(s)
	Phenylalanine dipeptide compounds			
				
	Lead compound			
1	N-[(N-benzoyl-L-phenylalanyl)-O-acetyl]-L-phenylalanol (MTS)	Whole plant	Ethanol extracts	Liang et al. (2002a); Liu et al. (2003)
	Derivatives containing ether structure			
2	Bentysrepinine	—	Based on MTS	Liang et al. (2002b)
3	N-[N-(4-propargyloxy benzoyl)-O-2-dimethylaminoethyl) -l-tyrosyl]-L-phenylalanol	—	Based on MTS	Yuan (2015)
4	N-[(N-benzoyl-L-phenylalanyl)-O-2-(dimethylaminoethyl) -l-tyrosyl]-O-propargyl-L-phenylalanol	—	Based on MTS	Yuan (2015)
5	N-[(2S)-1-{[(2S)-1-Hydroxy-3-phenylpropan-2-yl]amino}-3-(4-methoxyphenyl)-1-oxopropan-2-yl]benzamide	—	Based on MTS	Qiu et al. (2016)
6	N-[(2S)-3-(4-Ethoxyphenyl)-1-{[(2S)-1-hydroxy-3-phenylpropan-2-yl]amino}-1-oxopropan-2-yl]benzamide	—	Based on MTS	Qiu et al. (2016)
7	N-[(2S)-1-{[(2S)-1-Hydroxy-3-phenylpropan-2-yl]amino}-1-oxo-3-(4-propoxyphenyl)propan-2-yl]benzamide	—	Based on MTS	Qiu et al. (2016)
8	N-{(2S)-1-{[(2S)-1-Hydroxy-3-phenylpropan-2-yl]amino}-1-oxo-3-[4-(propan-2-yloxy)phenyl]propan-2-yl}benzamide	—	Based on MTS	Qiu et al. (2016)
9	N-[(2S)-3-(4-Butoxyphenyl)-1-{[(2S)-1-hydroxy-3-phenylpropan-2-yl]amino}-1-oxopropan-2-yl]benzamide	—	Based on MTS	Qiu et al. (2016)
10	N-{(2S)-1-{[(2S)-1-Hydroxy-3-phenylpropan-2-yl]amino}-3-[4-(2-methylpropoxy)phenyl]-1-oxopropan-2-yl} benzamide	—	Based on MTS	Qiu et al. (2016)
11	N-{(2S)-1-{[(2S)-1-Hydroxy-3-phenylpropan-2-yl]amino}-1-oxo-3-[4-(pentyloxy)phenyl]propan-2-yl}benzamide	—	Based on MTS	Qiu et al. (2016)
12	N-[(2S)-3-[4-(Hexyloxy)phenyl]-1-{[(2S)-1-hydroxy-3-phenylpropan-2-yl]amino}-1-oxopropan-2-yl]benzamide	—	Based on MTS	Qiu et al. (2016)
13	N-[(2S)-3-[4-(Cyanomethoxy)phenyl]-1-{[(2S)-1-hydroxy-3-phenylpropan-2-yl]amino}-1-oxopropan-2-yl]benzamide	—	Based on MTS	Qiu et al. (2016)
14	N-{(2S)-1-{[(2S)-1-Hydroxy-3-phenylpropan-2-yl]amino}-1-oxo-3-[4-(prop-2-en-1-yloxy)phenyl]propan-2-yl} benzamide	—	Based on MTS	Qiu et al. (2016)
15	(2S)-2-{[(2S)-2-Benzamido-3-(4-methoxyphenyl)propanoyl]amino}-3-phenylpropyl acetate	—	Based on MTS	Qiu et al. (2016)
16	(2S)-2-{[(2S)-2-Benzamido-3-(4-ethoxyphenyl)propanoyl] amino}-3-phenylpropyl acetate	—	Based on MTS	Qiu et al. (2016)
17	(2S)-2-{[(2S)-2-Benzamido-3-(4-propoxyphenyl)propanoyl]amino}-3-phenylpropyl acetate	—	Based on MTS	Qiu et al. (2016)
18	(2S)-2-({(2S)-2-Benzamido-3-[4-(propan-2-yloxy)phenyl] propanoyl}amino)-3-phenylpropyl acetate	—	Based on MTS	Qiu et al. (2016)
19	(2S)-2-{[(2S)-2-Benzamido-3-(4-butoxyphenyl)propanoyl] amino}-3-phenylpropyl acetate	—	Based on MTS	Qiu et al. (2016)
20	(2S)-2-({(2S)-2-Benzamido-3-[4-(2-methylpropoxy)phenyl]propanoyl}amino)-3-phenylpropyl acetate	—	Based on MTS	Qiu et al. (2016)
21	(2S)-2-({(2S)-2-Benzamido-3-[4-(pentyloxy)phenyl] propanoyl}amino)-3-phenylpropyl acetate	—	Based on MTS	Qiu et al. (2016)
22	(2S)-2-({(2S)-2-Benzamido-3-[4-(hexyloxy)phenyl] propanoyl}amino)-3-phenylpropyl acetate	—	Based on MTS	Qiu et al. (2016)
23	(2S)-2-({(2S)-2-Benzamido-3-[4-(cyanomethoxy)phenyl] propanoyl}amino)-3-phenylpropyl acetate	—	Based on MTS	Qiu et al. (2016)
24	(2S)-2-({(2S)-2-Benzamido-3-[4-(prop-2-en-1-yloxy) phenyl]propanoyl}amino)-3-phenylpropyl acetate	—	Based on MTS	Qiu et al. (2016)
	Fluorine or chlorine-substituted derivatives			
25	N-[N-(2-chlorobenzoyl)-l-tyrosyl]-L-phenylalanine methyl ester	—	Based on MTS	Kuang et al. (2019a)
26	N-[N-(4-fluorobenzoyl)-l-tyrosyl]-L-phenylalanine methyl ester	—	Based on MTS	Kuang et al. (2019a)
27	N-[N-(2-chlorobenzoyl)-l-tyrosyl]-L-phenylalanol	—	Based on MTS	Kuang et al. (2019a)
28	N-[N-(4-fluorobenzoyl)-l-tyrosyl]-L-phenylalanol	—	Based on MTS	Kuang et al. (2019a)
29	N-[N-(4-chlorobenzoyl)-l-tyrosyl]-L-phenylalanol	—	Based on MTS	Kuang et al. (2019a)
30	N-[N-(4-chlorobenzoyl)-O-methyl-l-tyrosyl]-L-phenylalanol	—	Based on MTS	Kuang et al. (2019a)
31	N-[N-(4-chlorobenzoyl)-O-ethyl-l-tyrosyl]-L-phenylalanol	—	Based on MTS	Kuang et al. (2019a)
32	N-[N-(4-chlorobenzoyl)-O-propyl-l-tyrosyl]-L-phenylalanol	—	Based on MTS	Kuang et al. (2019a)
33	N-[N-(4-chlorobenzoyl)-O-isopropyl-l-tyrosyl]-L-phenylalanol	—	Based on MTS	Kuang et al. (2019a)
34	N-[N-(4-chlorobenzoyl)-O-dimethylaminoethyl-l-tyrosyl]-L-phenylalanol	—	Based on MTS	Kuang et al. (2019a)
	Derivatives containing veratric acid			
35	N-[N-(3, 4-dimethoxy-benzoyl)-L-phenylalanyl]-L-tyrosine methyl ester	—	Based on MTS	Kuang et al. (2019b)
36	N-[N-(3, 4-dimethoxy-benzoyl)-l-tyrosyl]-L-phenylalanine methyl ester	—	Based on MTS	Kuang et al. (2019b)
37	N-[N-(3, 4-dimethoxy-benzoyl)-L-phenylalanyl]-L-phenylalanine methyl ester	—	Based on MTS	Kuang et al. (2019b)
38	N-[N-(3, 4-dimethoxy-benzoyl)-O-acetyl-l-tyrosyl] -L-phenylalanine methyl ester	—	Based on MTS	Kuang et al. (2019b)
39	N-[N-(3, 4-dimethoxy-benzoyl)-O-(2-diethyllaminoethyl)-l-tyrosyl]-L-phenylalanine methyl ester	—	Based on MTS	Kuang et al. (2019b)
40	N-[N-(3, 4-dimethoxy-benzoyl)-O-ethyl-l-tyrosyl]-L-phenylalanine methyl ester	—	Based on MTS	Kuang et al. (2019b)
41	N-[N-(3, 4-dimethoxy-benzoyl)-O-propyl-l-tyrosyl]-L-phenylalanine methyl ester	—	Based on MTS	Kuang et al. (2019b)
42	N-[N-(3, 4-dimethoxy-benzoyl)-L-phenylalanyl-O-ethyl]-L-tyrosine methyl ester	—	Based on MTS	Kuang et al. (2019b)
43	N-[N-(3, 4-dimethoxy-benzoyl)-L-phenylalanyl-O-propyl] -L-tyrosine methyl ester	—	Based on MTS	Kuang et al. (2019b)
44	N-[N-(3, 4-dimethoxy-benzoyl)-L-phenylalanyl-O-isopropyl] -L-tyrosine methyl ester	—	Based on MTS	Kuang et al. (2019b)
45	N-[N-(3, 4-dimethoxy-benzoyl)-L-phenylalanyl-O-n-butyl] -L-tyrosine methyl ester	—	Based on MTS	Kuang et al. (2019b)
46	N-[N-(3,4-dimethoxy-benzoyl)-L-phenylalanyl-O-n-octyl]-L-tyrosine methyl ester	—	Based on MTS	Kuang et al. (2019b)
47	N-[N-(3, 4-dimethoxy-benzoyl)-L-phenylalanyl]-L -phenylalanol	—	Based on MTS	Kuang et al. (2019b)
48	N-[N-(3, 4-dimethoxy-benzoyl)-L-phenylalanyl]-L-tyrosinol	—	Based on MTS	Kuang et al. (2019b)
49	N-[N-(3, 4-dimethoxy-benzoyl)-L-phenylalanyl]-O-acetyl-L-phenylalanol	—	Based on MTS	Kuang et al. (2019b)
50	N-[N-(3, 4-dimethoxy-benzoyl)-L-phenylalanyl]-O-propionyl-L-phenylalanol	—	Based on MTS	Kuang et al. (2019b)
51	N-[N-(3, 4-dimethoxy-benzoyl)-L-phenylalanyl]-4-ethoxy-L-phenylalanol	—	Based on MTS	Kuang et al. (2019b)
52	N-[N-(3, 4-dimethoxy-benzoyl)-L-phenylalanyl]-4-e*thoxycarbonylmethyl*-L-tyrosinol	—	Based on MTS	Kuang et al. (2019b)
53	N-[N-(3, 4-dimethoxy-benzoyl)-L-phenylalanyl] -O-acetyl-(O-acetyl)-L-tyrosinol	—	Based on MTS	Kuang et al. (2019b)
54	N-[N-(3, 4-dimethoxy-benzoyl)-L-phenylalanyl]-4-propionyloxy-O-propionyl -L-phenylalanol	—	Based on MTS	Kuang et al. (2019b)
	Ester derivatives			
55	N-[N-benzoyl-O-(2-dimethylaminoethyl)-l-tyrosyl] -O-(4-fluorobenzoyl)-L-phenylalanol	—	Based on MTS	Liang et al. (2013)
56	N-[N-benzoyl-O-(2-dimethylaminoethyl)-l-tyrosyl] -O-(4-chlorobenzoyl)-L-phenylalanol	—	Based on MTS	Liang et al. (2013)
57	N-[N-benzoyl-O-(2-dimethylaminoethyl)-l-tyrosyl] -O-(4-methylbenzoyl)-L-phenylalanol	—	Based on MTS	Liang et al. (2013)
58	N-[N-benzoyl-O-(2-dimethylaminoethyl)-l-tyrosyl] -O-(2-methoxybenzoyl)-L-phenylalanol	—	Based on MTS	Liang et al. (2013)
59	N-[N-benzoyl-O-(2-dimethylaminoethyl)-l-tyrosyl] -O-(3-trifluoromethoxybenzoyl)-L-phenylalanol	—	Based on MTS	Liang et al. (2013)
60	N-[N-benzoyl-O-(2-dimethylaminoethyl)-l-tyrosyl] -O-(3-bromobenzoyl)-L-phenylalanol	—	Based on MTS	Liang et al. (2013)
61	N-[N-benzoyl-O-(2-dimethylaminoethyl)-l-tyrosyl] -O-(2-methylbenzoyl)-L-phenylalanol	—	Based on MTS	Liang et al. (2013)
62	N-[N-benzoyl-O-(2-dimethylaminoethyl)-l-tyrosyl] -O-(2-furoyl)-L-phenylalanol	—	Based on MTS	Liang et al. (2013)
63	N-[N-benzoyl-O-(2-dimethylaminoethyl)-l-tyrosyl] -O-(2-thenoyl)-L-phenylalanol	—	Based on MTS	Liang et al. (2013)
64	N-[N-benzoyl-O-(2-dimethylaminoethyl)-l-tyrosyl] -O-(4-picolinoyl)-L-phenylalanol	—	Based on MTS	Liang et al. (2013)
65	N-[N-benzoyl-O-(2-dimethylaminoethyl)-l-tyrosyl] -O-(3-picolinoyl)-L-phenylalanol	—	Based on MTS	Liang et al. (2013)
	Derivatives with aromatic heterocycles			
66	N-[N-(2-furoyl)-L-phenylalanyl]-L-tyrosine methyl ester	—	Based on MTS	Qiu et al. (2015)
67	N-[N-nicotinoyl-L-phenylalanyl]-L-tyrosine methyl ester	—	Based on MTS	Qiu et al. (2015)
68	N-[N-nicotinoyl-L-phenylalanyl]-L-phenylalanine methyl ester	—	Based on MTS	Qiu et al. (2015)
69	N-[N-nicotinoyl-L-phenylalanyl]-L-tyrosine	—	Based on MTS	Qiu et al. (2015)
70	N-[N-nicotinoyl-L-phenylalanyl]-O-acetyl-tyrosine methyl ester	—	Based on MTS	Qiu et al. (2015)
71	N-[N-(2-furoyl)-O-(2-dimethylaminoethyl)-l- tyrosyl]-L-phenylalanol	—	Based on MTS	Qiu et al. (2015)
72	N-[N-(2-furoyl)-O-(2-diethyllaminoethyl)-l-tyrosyl]-L-phenylalanol	—	Based on MTS	Qiu et al. (2015)
73	N-[N-nicotinoyl-O-(2-dimethylaminoethyl)-l-tyrosyl]-L-phenylalanol	—	Based on MTS	Qiu et al. (2015)
74	N-[N-nicotinoyl-O-(2-diethyllaminoethyl)-l-tyrosyl]-L-phenylalanol	—	Based on MTS	Qiu et al. (2015)
75	N-[N-(2-furoyl)-O-acetoxy-l-tyrosyl]-L-phenylalanol	—	Based on MTS	Qiu et al. (2015)
76	N-(N-nicotinoyl-O-acetoxy-l-tyrosyl)-L-phenylalanol	—	Based on MTS	Qiu et al. (2015)
	Derivatives with nitrogen-containing heterocycles			
77	N-[N-benzoyl)-L-(5-bromonicotinoy)-O-acetoxy -acetoxy]-L-phenylalanine methyl ester	—	Based on MTS	Qiu et al. (2015)
78	N-[N-benzoyl)-L-(5-bromonicotinoy)-O-acetoxy -acetoxy]-L-phenylalaninol	—	Based on MTS	Qiu et al. (2015)
79	N-[N-benzoyl)-L-(5-bromonicotinoy)-O-acetoxy -acetoxy]-L-phenylalanine	—	Based on MTS	Qiu et al. (2015)
80	N-[N-benzoyl)-L-(5-bromonicotinoy)-O-acetoxy -acetoxy]-L-methyl chloride	—	Based on MTS	Qiu et al. (2015)
81	N-[N-benzoyl)-L-(5-bromonicotinoy)-O-acetoxy -acetoxy]-L-methyl acetate	—	Based on MTS	Qiu et al. (2015)
82	N-[N-benzoyl)-L-(5-bromonicotinoy)-O-acetoxy -acetoxy]-L-methyl propanoate	—	Based on MTS	Qiu et al. (2015)
	Derivatives containing trifluoromethyl			
83	N-[N-(3-trifluoromethylbenzoyl)-L-phenylalanyl]-L-phenylalanol	—	Based on MTS	Cui et al., 2019
84	N-[N-(3-trifluoromethylbenzoyl)-l-tyrosyl]-L-phenylalanol	—	Based on MTS	Qiu et al. (2015)
85	N-[N-(3-trifluoromethylbenzoyl)-L-phenylalanyl]-L-tyrosine methyl ester	—	Based on MTS	Qiu et al. (2015)
86	N-[N-(3-trifluoromethylbenzoyl)-l-tyrosyl]-L-tyrosine methyl ester	—	Based on MTS	Qiu et al. (2015)
87	N-[N-(3-trifluoromethylbenzoyl)-L-phenylalanyl]-L-phenylalanine methyl ester	—	Based on MTS	Qiu et al. (2015)
88	N-[N-(3-trifluoromethylbenzoyl)-l-tyrosyl]-L-phenylalanine methyl ester	—	Based on MTS	Qiu et al. (2015)
89	N-[N-(3-trifluoromethylbenzoyl)-L-phenylalanyl]-O-acetyl-L-phenylalanol	—	Based on MTS	Qiu et al. (2015)
90	N-[N-(3-trifluoromethylbenzoyl)-L-phenylalanyl]-O-propionyl-L-phenylalanol	—	Based on MTS	Qiu et al. (2015)
91	N-[N-(3-trifluoromethylbenzoyl)-L-phenylalanyl]-O-acetyl-L-tyrosine methyl ester	—	Based on MTS	Qiu et al. (2015)
92	N-[N-(3-trifluoromethylbenzoyl)-L-phenylalanyl]-O-propionyl-L-tyrosine methyl ester	—	Based on MTS	Qiu et al. (2015)
93	N-[N-(3-trifluoromethylbenzoyl)-O-(2-dimethylaminoethyl)-l-tyrosyl]-L-phenylalanol	—	Based on MTS	Qiu et al. (2015)
94	N-[N-(3-trifluoromethylbenzoyl)-O-(2-diethylaminoethyl)-l-tyrosyl]-L-phenylalanol	—	Based on MTS	Qiu et al. (2015)
95	N-[N-(3-trifluoromethylbenzoyl)-O-carboxymethyl-l-tyrosyl]-L-phenylalanol	—	Based on MTS	Qiu et al. (2015)
96	N-[N-(3-trifluoromethylbenzoyl)-L-phenylalanyl]-O- ethoxycarbonylmethyl-L-tyrosine methyl ester	—	Based on MTS	Qiu et al. (2015)
97	N-[N-(3-trifluoromethylbenzoyl)-L-phenylalanyl]-O-methyl -L-tyrosine methyl ester	—	Based on MTS	Qiu et al. (2015)
98	N-[N-(3-trifluoromethylbenzoyl)-L-phenylalanyl]-O-ethyl -L-tyrosine methyl ester	—	Based on MTS	Qiu et al. (2015)
99	N-[N-(3-trifluoromethylbenzoyl)-L-phenylalanyl]-O-propyl -L-tyrosine methyl ester	—	Based on MTS	Qiu et al. (2015)
100	N-[N-(3-trifluoromethylbenzoyl)-L-phenylalanyl]-L-phenylalanine	—	Based on MTS	Qiu et al. (2015)
101	N-[N-(3-trifluoromethylbenzoyl)-L-phenylalanyl]-O-methyl -L-tyrosine	—	Based on MTS	Qiu et al. (2015)
102	N-[N-(3-trifluoromethylbenzoyl)-L-phenylalanyl]-O-ethyl -L-tyrosine	—	Based on MTS	Qiu et al. (2015)
	Derivatives containing sulfonamide structure			
103	N-[N-(*p*-toluenesulfonyl)-L-phenylalanyl]-L-tyrosine methyl ester	—	Based on MTS	Xu et al. (2014)
104	N-[N-(*p*-toluenesulfonyl)-L-phenylalanyl]-L-phenylalanine methyl ester	—	Based on MTS	Xu et al. (2014)
105	N-(N-phenylsulfonyl-L-phenylalanyl)-L-phenylalanine methyl ester	—	Based on MTS	Xu et al. (2014)
106	N-(N-phenylsulfonyl-L-phenylalanyl)-L-phenylalanol	—	Based on MTS	Xu et al. (2014)
107	N-(N-benzenesulfonyl-L-phenylalanyl)-L-tyrosine methyl ester	—	Based on MTS	Xu et al. (2014)
108	N-[N-(*p*-toluenesulfonyl)-L-phenylalanyl]-L-phenylalanine	—	Based on MTS	Xu et al. (2014)
109	N-[N-octyl-(*p*-toluenesulfonyl)-L-phenylalanyl)]-O-(n-octyl) -tyrosine methyl ester	—	Based on MTS	Xu et al. (2014)
	Resin glycosides			
110	(11S)-dihydroxyhexadecanoic acid 11-O-(3-O-(2R,3R)-3 -hydroxy-2-methybutyryl)-α-l-rhamnopyranosyl-(1→4)-O-β-d-glucopyranose-(1→2)-[(2-O-(2R,3R)-3-hydroxy-2-methybutyryl)-α-l-rhamnopyranosyl-(1→6)]-O-β-d -glucopyranose (dichondrin A)	Whole plant	Acetone–H_2_O (4:1, v/v) extracts	Song et al. (2015)
111	(3S,11S)-dihydroxyhexadecanoic acid 11-O-(3-O-(2R,3R)-3 -hydroxy-2-methybutyryl)-α-l-rhamnopyranosyl-(1→4)-O-β-d-glucopyranose-(1→2)-[(2-O-(2R,3R)-3-hydroxy -2-methybutyryl)-α-l-rhamnopyranosyl-(1→6)]-O-β-d -glucopyranose (dichondrin B)	Whole plant	Acetone–H_2_O (4:1, v/v) extracts	Song et al. (2015)
112	α-l-rhamnopyranosyl-(1→3)-[1-O-(11S)-11-hydroxyhexadecanoyl]-[4-O-(2R,3R)-3-hydroxy-2-methybutyryl]-α-l-rhamnopyranosyl-(1→2)-O-d -glucopyranose (dichondrin C)	Whole plant	Acetone–H_2_O (4:1, v/v) extract	Song et al. (2015)
113	Resin glycoside cus-1	Whole plant	Acetone–H_2_O (4:1, v/v) extract	Song et al. (2015)
114	Resin glycoside cus-2	Whole plant	Acetone–H_2_O (4:1, v/v) extract	Song et al. (2015)
115	Resin glycoside cuse 3	Whole plant	Acetone–H_2_O (4:1, v/v) extract	Song et al. (2015)
	Terpenoids			
116	Tormentic acid	Whole plant	n-BuOH extract	Liu et al. (2002); Liu et al. (2003)
117	Ursolic acid	Whole plant	n-BuOH extract	Liu et al. (2002); Liu et al. (2003)
118	(2E, 6E)-8,10,11-trihydroxyl-7,11-dimethyl-3-hydroxymethyl-2,6-dodecadienoic acid	Whole plant	H_2_O/acetone extract (2:8, v/v)	Wang and Xuan (2015)
119	(2E,6E)-8,10,11-trihydroxyl-7,11-dimethyl-3-hydroxymethyl-2,6-dodecadienoic acid 13-O-β-d-glucopyranoside	Whole plant	H_2_O/acetone extract	Wang and Xuan (2015)
120	6,6-Dimethyl-2-methlenebicyclo [3.1.1]hept-3-O-(6-O-apiofuranosyl)-β-D-gluco-pyranoside	Whole plant	H_2_O/acetone extract	Wang and Xuan (2015)
	Coumarins			
121	Skimmin	Whole plant	n-BuOH extract	Liu et al. (2002)
122	Scopoletin	Whole plant	n-BuOH extract	Liu et al. (2002)
123	Umbelliferone	Whole plant	n-BuOH extract	Liu et al. (2002)
124	Uracils	Whole plant	n-BuOH extract	Liu et al. (2003)
125	Uracil Steroids β-sitosterol	Whole plant	n-BuOH extract	Liu et al. (2003)

**TABLE 4:** 35 volatile oil components isolated and identified from DRF.

No	Compound name	No	Compound name
1	2-Pentylfuran	19	*trans*-caryophyllene
2	Limonene	20	5-Epi-aristolochene
3	*trans*-β-ocimene	21	β-selinene
4	*trans*-ocimene	22	Isoledene
5	Terpinolene	23	Eremophilene
6	Linalool	24	β-chamigrene
7	*trans*-pinocarveol	25	Junipene
8	*p*-vinylanisole	26	δ-cadinene
9	[+]-α-terpineol	27	α-calacorene
10	Myrtenol	28	d-nerolidol
11	*trans*-geraniol	29	2-Tetradecanone
12	α-citral	30	Spathulenol
13	α-cubebene	31	Caryophyllene oxide
14	cyclo-isosativene	32	Heptadecane
15	Copaene	33	6, 10, 14-trimethy1-2-pentadecanone
16	*trans*-β-damascenone	34	Tetradecanal
17	β-elemene	35	n-hexadecanoic acid
18	*cis*-caryophyllene		

**TABLE 5:** 31 volatile oil components isolated and identified from DRF.

No	Compound name	No	Compound name
1	2-Methoxyphenol	17	(-)-Spathulenol
2	3,7-Dimethyl-1,6-octadien-3-ol	18	4-(2,2-Dimethyl-6-methylenecyclohexyl)-2-butanone
3	[+]-α-terpineol	19	*cis*-9-Tetradecen-1-ol
4	*trans*-geraniol	20	bicyclo [3.2.2]non-6-en-3-one
5	2,6-Dimethoxyphenol	21	*trans*-Z-α-bisabolene epoxide
6	α-Cubebene	22	2,6,6-Trimethyl-(1α,2β,5α)-bicyclo [3.1.1] Heptane
7	Isocaryophyllene	23	Heptadecane
8	Caryophyllene	24	2-methyl-Z-4-tetradecene
9	β-Humulene	25	Methyl ester hexadecanoic acid, 2-hydroxy-
10	1,13-Tridecanediol, diacetate	26	6-(methylamino)phenanthren-3-ol
11	(-)-trans-Pinane	27	6-Octadecenal
12	naphthalene,1,2,3,5,6,7,8,8a-octahydro-1,8a-Dimethyl-7 (1-methenyl)-, [15-(1α,7α, 8a α0)-	28	*cis*-4-Hydroxy-3-methylundecanoic acid
13	naphthalene,1,2,3,5,6,7,8,8a-octahydro-1,8a-Dimethyl-7 (1-methenyl)-, [1R-(1α,7α, 8a α0)-ylangene	29	1H-cycloprop [e]azulene, decahydro-1,1,7-Trimethyl-4-methylene
14	2-Isopropenyl-4a,8-dimethyl-1,2,3,4,4a,5,6,8a-Octahydronaphthalene	30	Lactone
15	3,7,7-Trimethyl-11-methylene-, (-)-spiro [5.5] Undec-2-ene	31	Phytol
16	(+)-cis-nerolidol		

### Phenylalanine Dipeptide Compounds

MTS (chemical name [N-(N-benzoyl-L-phenylalanyl)-O-acetyl-L-phenylalanol]) (1) is a key phenylalanine dipeptide compound and a characteristic substance isolated from DRF (Liang et al., 2002a; Liu et al., 2003). However, no study on MTS has been carried out in DRF samples from different production areas regarding seasons and parts of the plant. Recently, *ß*-sitosterol was selected as a control to distinguish DRF samples from different production areas of Guangxi province by using thin layer chromatography (TLC) (Lan, 2013). In addition, umbelliferone content in DRF was determined by using high performance liquid chromatography (HPLC) at 325 nm (Liao and Pan, 2013). In summary, these methods above provided good references to control the quality of DRF by detecting some non-characteristic substance such as *ß*-sitosterol or characteristic component like umbelliferone in this plant.

After that, a series of derivatives including 24 derivatives containing ether structure (2～24) (Xu et al., 2009; Yuan, 2015; Qiu et al., 2016), 10 fluorine or chlorine-substituted derivatives (25～34) (Kuang et al., 2019a), 20 derivatives containing veratric acid (35～54) (Kuang et al., 2019b), 11 ester derivatives (55～65) (Liang et al., 2013), 11 derivatives with aromatic heterocycles (66～76) (Qiu et al., 2015), 10 derivatives with nitrogen-containing heterocycles (77～82) (Jiang et al., 2019), 20 derivatives containing trifluoromethyl (83～102) (Cui et al., 2019), and seven derivatives containing sulfonamide structure (103～109) (Xu et al., 2014) were synthesized based on the lead compound MTS. These compounds were then identified by nuclear magnetic resonance (NMR) and electrospray ionization mass spectrometry (ESI-MS). *In vitro* activity tests revealed some derivatives of MTS had good anti-hepatitis B virus activities. Their chemical names and structures are shown in Table 3 and Figure 2.

### Resin Glycosides

Resin glycosides are primarily found in the family Convolvulaceae. They are unusual amphipathic metabolites, containing structures with hydrophilic (oligosaccharide) and hydrophobic (fatty acid aglycone) moieties.

Currently, three resin glycosides named dichondrins A-C (110～112) and three known resin glycosides cus-1, cus-2, and cus-3 (113～115) have been isolated from DRF. Further, their structures were elucidated by the results of high resolution electrospray ionization mass spectroscopy (HRESIMS), ^1^H, and ^13^C NMR data (Song et al., 2015). Their chemical names and structures are listed in Table 3 and Figure 2.

### Terpenoids

Terpenoids are compounds derived from mevalonic acid and their molecular skeletons have isoprene units (C5 units) as the basic structural units. Terpenoids are widely present in nature, and they are main constituents of fragrances, resins, and pigments.

Currently, two triterpenoid compounds including tormentic acid (116) and ursolic acid (117) were isolated from the whole plant of DRF and then identified by infrared spectrum (IS), mass spectrum (MS), NMR and TLC (Liu et al., 2002; Liu et al., 2003). In addition, a new highly oxygenated acyclic sesquiterpenoid (118) and its glucoside (119), together with a new pinane monoterpene (120) have been separated from DRF. Subsequently, the structures of these three compounds were elucidated by spectroscopic analyses and chemical methods (Wang and Xuan 2015). Their chemical names and structures are displayed in Table 3 and Figure 2.

### Coumarins

Coumarins are a general term for O-hydroxycinnamic acid lactones. The core of coumarin is phenyl *a*-pyrone. The ring often has substituents such as hydroxyl, alkoxy, phenyl and isopentenyl. Among them, the active double bond of isopentenyl and the ortho hydroxyl in the benzene ring can form a furan ring or a pyran ring structure. Coumarins are a class of natural products with strong biological activities including anti-coagulation, anti-tumor, anti-virus, enhancing autoimmunity, anti-proliferation, anti-AIDS, and anti-fatigue.

Three coumarin compounds such as skimmin (121), scopoletin (122) and umbelliferone (123) were isolated from the whole plant of DRF. Their chemical structures were identified by IS, MS, and NMR (Liu et al., 2002). Their chemical names and structures are listed in Table 3 and Figure 2.

### Flavonoids

It has been reported that various flavonoid compounds including flavonoids, flavonols, isoflavones are isolated from DRF (Yang et al., 2005). However, the detail information on chemical names and structures of these compounds remains unknown.

### Uracil Compounds

Uracil (124) has been separated from the whole plant of DRF (Liu et al., 2003), and its corresponding chemical name and structure are shown in Table 3 and Figure 2.

### Steroids

β-sitosterol (125) has been separated from the whole plant of DRF (Liu et al., 2002), and its chemical name and structure are displayed in Table 3 and Figure 2.

### Volatile Oils

Liang (Liang et al., 2002b) isolated and identified 35 chemical components which were mainly monoterpenes, sesquiterpenes and their oxygenated derivatives including trans-caryophyllene and iso-cadinene by using gas chromatograph (GC) in combination with MS (Table 4). In addition, a total of 31 essential oil compositions were identified from DRF by gas chromatograph-mass spectrum (GC-MS) (Table 5). The top three compounds identified included 6-(methylamino)-phenanthren-3-ol (–)-trans-pinane, and ylangene (Wu et al., 2009). Among these volatile oil components, the repeated ones were [+]-α-terpineol, *trans*-geraniol, *a*-cubebene, and heptadecane.

## Pharmacological Effects

Pharmacological effects have been conducted in compounds and crude extracts derived from DRF. The pharmacological effects are outlined in Table 6.

**TABLE 6:** Pharmacological effects of DRF.

Effects	Active components/compounds	Source	References(s)
Anti-HBV effects	MTS (1)	Whole plant	Liang et al. (2002a)
	bentysrepinine (2)	Synthesis based on MTS	Xu et al. (2009)
	MTS derivatives containing ether structure (11, 14, 17, 21～23)	Synthesis based on MTS	Qiu et al. (2016)
	Fluorine or chlorine-substituted derivatives (30, 32, 33)	Synthesis based on MTS	Kuang et al. (2019a)
	Derivatives containing veratric acid (50～52)	Synthesis based on MTS	Kuang et al. (2019b)
	Derivatives with aromatic heterocycles (71, 75)	Synthesis based on MTS	Qiu et al. (2015)
	Derivatives with nitrogen-containing heterocycles (79, 81, 82). MTS derivatives containing trifluoromethyl (84, 89, 91, 92, 96～98, 102). MTS derivatives containing sulfonamide structure (104, 106, 108)	Synthesis based on MTS	Jiang et al. (2019); Cui et al. (2019); Xu et al. (2014)
Anti-inflammatory and antioxidant effects	Ethanol extracts of DRF. n-butanol extracts of DRF. Petroleum ether extracts of DRF	Whole plant	Sheu et al. (2012); Qu et al. (2003a); Zeng et al. (2005)
Antipyretic effects	n-butanol extracts of DRF	Whole plant	Qu et al. (2003b)
Analgesic effects	n-butanol extracts of DRF	Whole plant	Qu et al. (2003a)
	Petroleum ether extracts of DRF	Whole plant	Zeng et al. (2005)
Antibacterial effects	n-butanol extracts of DRF	Whole plant	Qu et al. (2003a)
	Essential oils of DRF	Whole plant	Wu et al. (2009)
Anti-tumor effects	MTS derivatives (6～8, 10, 14, 15, 24)	Synthesis based on MTS	Qiu et al. (2016)
	Dichondrins C (112), resin glycosides cus-3 (115)	Acetone-H_2_O (4:1,v/v) extract	Song et al. (2015)
Hepatoprotective effects	n-butanol extracts of DRF	Whole plant	Qu et al. (2003c), Qu et al. (2003d); Zeng et al. (2011)
Cholagogic effects	n-butanol extracts of DRF	Whole plant	Qu et al. (2003b)
Immunomodulatory effects	n-butanol extracts of DRF	Whole plant	Qu et al. (2003b)

### Anti-HBV Effects

Hepatitis B virus (HBV) is a pathogen that causes hepatitis B, characterized by anorexia, nausea, upper abdominal discomfort, and hepatalgia in clinic, threatening people's health seriously.

Anti-HBV effects of DRF are based on its heat-clearing and removing the phlegm and turbid urine effects (The minister of health of logistics department of Kunming military command, 1970; Guangxi health administration, 1970; Fujian institute of medicine, 1970). Some derivatives of MTS exhibit strong anti-HBV activities as follows.

#### Derivatives Containing Ether Structure

Among the derivatives containing ether structure, compound (2) named bentysrepinine (Y101, Chinese name Tifentai) showed an excellent anti-HBV activity, and its phase I clinical trial (CFDA registration number CTR20160,096) has been completed.

*In vitro*, anti-HBV activity of bentysrepinine was determined in HepG2.2.15 cells (HepG two cell lines transfected with HBV gene stably) by using 3-(4,5-dimethyl -2-thiazolyl)-2,5-diphenyl-2-H-tetrazolium bromide (MTT) method (Kuang et al., 2019a). Briefly, various concentrations of bentysrepinine were respectively cocultured with the HepG2.2.15 cells in a 96-well plate for 72 h. Lamivudine was selected as a positive control. After that, MTT solution (5 mg/ml) was added for another 6 h. Subsequently, 200 μL of DMSO was added to solve the formazan and OD value was measured at 570 nm to detect the cell viability. In addition, polymerase chain reaction (PCR)-fluorescent probe was used to detect the DNA loads. The results revealed that bentysrepinine had no significant cytotoxicity at or under 50 μg/ml. After treated with bentysrepinine for 3 and 6 days, copy number of HBV-DNA in the cells was decreased in a concentration-dependent manner. Compared with the control, at the sixth day bentysrepinine (12.5, 25 and 50 μg/ml) remarkably reduced HBV-DNA loads with average inhibition rates of 56.57%, 62.83% and 79.09%, respectively (Wang and Xuan 2015). The inhibitory effect of bentysrepinine on hepatitis B surface antigen (HBsAg) was also determined. The HepG2.2.15 cells were treated with various concentrations of bentysrepinine (102, 51 and 25.5 μmol/L) in a 24-well plate for 8 days and the media were replaced every 4 days. The cell supernatants were collected on the eighth day and the HBsAg titer was measured by using ELISA. The results indicated that bentysrepinine remarkedly reduced the HBsAg titer in a concentration-dependent manner with the average inhibition rates of 56.26, 47.33, and 36.82%, respectively. The results suggested that bentysrepinine had a strong inhibitory effect on HBV-DNA.

Further, Sun investigated the anti-HBV mechanism of bentysrepinine by using gene chip (Sun et al., 2019) in HepG2 A64 cells (both lamivudine- and entecavir-resistant HepG2 cell lines with mutants in rtL180M plus rtM204V plus rtT184 L). The results demonstrated that compared with the control group, expressions of 11 genes in bentysrepinine-treated group were changed by more than 10 folds, including ZNF503-AS1, SPINK4, IL-21R, CIB4, GPNMB, CELF2-AS2, ACP5, KLHDC7B, SYT4, MRPL23-AS1; KEGG analysis revealed nine genes with over 1.5-fold change, including LAMB3, TNF, p21, bcl-2, CDK6, CERB, PKC, Pyk2 and FOS. These nine differential genes participate in the regulation of HBV infection-related molecule networks, thereby interfering the transduction of intracellular Calcium-Pyk2 pathway, which enables bentysrepinine to exert its anti-HBV effects. These findings suggested that bentysrepinine regulated Calcium-Pyk2 pathway and p21 at the downstream of HBV copy, influencing function of HBx protein, which finally inhibited the copy of HBV. In addition, bentysrepinine regulated immune factors such as IL-21 and Bcl-2, which enhanced cellular anti-HBV capability.

Other MTS derivatives containing ether structure including compounds (11, 14, 17, 21～23) exhibited inhibitory effects on HBV DNA replication in the HepG2 2.2.15 cells with the IC_50_ values between 2.18 and 8.55 μmol/L, which was much lower than lamivudine, the positive control (IC_50_ = 82.42 μmol/L) (Qiu et al., 2016). In particularly, the compounds (22) (IC_50_ = 2.18 μmol/L; selectivity index (SI) = 151.59) and (24) (IC_50_ = 5.65 μmol/L; SI = 51.16) had low cytotoxicity and high SI values. Unexpectedly, the compound (14) not only markedly inhibited activity of HBV DNA replication, but also significantly suppressed proliferation of two hepatocellular carcinoma cell (HCC) lines QGY-7701 and SMMC-7721 provided by ATCC (Qiu et al., 2016), suggesting it might be a promising lead drug to treat HBV infection and HBV-related HCC in the future.

#### Fluorine or Chlorine-Substituted Derivatives

Anti-HBV activities of MTS fluorine or chlorine-substituted derivatives including compounds (25～34) were determined in the HepG2.2.15 cells by the MTT method. The modified Käber method was used to calculate TC_50_ values of the drugs. In addition, the cells were treated with these above drugs respectively. Then the culture media containing these drugs with different dilution concentrations were replaced every 72 h. The cells were collected at the eighth day after the treatments. The HBV DNA replication in the cells was detected by dot blot hybridization, and IC_50_ and selective index (SI) were calculated. The results revealed that compounds (30), (32) and (33) showed good anti-HBV activities with IC_50_ values of 12.60, 10.50, 6.46 μmol/L, respectively (Kuang et al., 2019a). Among these four compounds, the compound (30) also had slightly reduced cytotoxicity and SI (TC_50_/IC_50_) value of 53.0.

#### Derivatives Containing Veratric Acid

The MTT method was used to test anti-HBV activities of 20 MTS derivatives containing veratric acid (35～54) in the HepG2.2.15 cells. Seven compounds were found to exhibit inhibitory effect on HBV DNA at the concentrations tested. Among them, the compounds (50～52) had better anti-HBV activities (IC_50_ = 6.17, 9.18 and 8.29 μmol/L, respectively) than MTS and lamivudine (Kuang et al., 2019b).

#### Derivatives With Aromatic Heterocycles

Anti-HBV activity test was conducted in 11 MTS derivatives with aromatic heterocycles (66–76) in the HepG2.2.15 cells. The results demonstrated that the compounds (71) (IC_50_ = 2.94 μmol/L, SI = 146.39) and (75) (IC_50_ = 2.21 μmol/L, SI > 250) showed strong inhibitory activities against HBV and significantly inhibited the HBV DNA replication compared with the lead compound MTS (IC_50_ = 11.16 μmol/L, SI = 10.78) (Qiu et al., 2015). It suggested that these two compounds had higher safety in suppressing the HBV replication and were potential in the future research on anti-HBV infections.

#### Derivatives With Nitrogen-Containing Heterocycle

6 MTS derivatives with nitrogen-containing heterocycles (77～82) were used for the anti-HBV activity test in the HepG2.2.15 cells (Jiang et al., 2019). The replication of HBV DNA, IC_50_ and SI were detected. The results displayed that the compounds (79, 81, 82) had significant inhibitory effects on the HBV copy (IC_50_ = 8.73, 15.26, 37.15 μmol/L and SI = 53.68, 19.43, 10.02, respectively). Further, the compound (79) exhibited a better anti-HBV activity, suggesting the role of nitrogen-containing heterocycles substitution in the anti-HBV test based on the prototype MTS.

#### Derivatives Containing Trifluoromethyl

Anti-HBV activity was detected in 20 MTS derivatives containing trifluoromethyl (83～102) in the HepG2.2.15 cells described as before (Cui et al., 2019). The activity test results indicated that the compounds (84, 89, 91, 92, 96～98, 102) showed certain inhibitory effects on the HepG2 2.2. 15 cells, of which the compounds (84, 92, 102) had strong anti-HBV activities with the IC_50_ values of 11.74, 8.73 and 11.41 μmol/L, respectively.

#### Derivatives Containing Sulfonamide Structure

7 MTS derivatives containing sulfonamide structure (103～109) were selected to test their anti-HBV activities (Xu et al., 2014). The results demonstrated that the compounds (104, 106, 108) showed strong anti-HBV activities at a same concentrations of 8 μg/ml with the inhibition rates of 41.9, 61.3, and 47.5%, respectively. In addition, the compound (106) significantly inhibited HBV at a concentration of 3.2 μg/ml, with an inhibition rate of 50.9%.

In summary, although MTS and its some derivatives displayed anti-HBV activities *in vitro*, besides bentysrepinine, until now few related research has been documented on their anti-HBV activities in HBV infection-related animal models. Further, the mechanisms for anti-HBV of MTS and its derivatives still remain unknown. Thus, it is necessary to do some works for *in vivo* anti-HBV studies and action mechanisms of these phenylalanine dipeptide compounds derived from DRF.

### Anti-inflammatory and Antioxidant Effects

Heat-clearing effects of DRF are based on its anti-inflammatory effects (The minister of health of logistics department of Kunming military command, 1970; Guangxi health administration, 1970; Fujian institute of medicine, 1970). Ethanol extracts of DRF (EDR) displayed antioxidant and anti-inflammatory effects by regulating pro-inflammatory mediators such as NO, iNOS and COX2 and pro-inflammatory cytokine such as TNF-α (Sheu et al., 2012). It indicated that the anti-inflammatory and antioxidant effects of DRF are related to the suppressions on some related molecules including TNF-α, NO, and COX2.

Sheu et al. (2012) evaluated anti-inflammatory and antioxidant effects of EDR *in vitro* and *in vivo*. *In vitro*, antioxidant effects of EDR (125, 250, 500, and 1,000 μg/ml) were assessed by using ABTS radical-scavenging test, DPPH radical-scavenging test, ferric reducing antioxidant property (FRAP), lipid peroxidation assay and total polyphenol content assay in LPS (100 ng/ml)-stimulated RAW264.7 cells. *In vivo*, a λ-Carrageenan (Carr)-induced edema model was used to evaluate anti-inflammatory activity of EDR. After the inflammations, the paw volumes were recorded within 5 h and once 1 h post the Carr injections by using a plethysmometer. The degree of Carr-induced edema was calculated by ratio of volume of right hind paw after and before the injection. At the end of the experiment, the right hind paw tissue and liver tissue were dissected and homogenized at 4°C. The homogenate was centrifuged at 12,000 ×*g* for 5 min. The supernatant was harvested for MDA assay. The whole liver tissue was rinsed and homogenized at 4°C. Then, the homogenate was centrifuged and the supernatant was collected for CAT, SOD, and GPx activity assays. The sera were collected for NO and TNF-a assays by using ELISA. The results showed that EDR reduced the LPS-induced NO production and expressions of iNOS and COX-2 in the LPS-stimulated RAW 264.7 cells. *In vivo*, EDR significantly attenuated Carr-induced paw edema and suppressed the serum levels of TNF-a and NO. In addition, EDR markedly decreased the MDA content in the edema paw and significantly increased the activities of CAT, SOD and GPx in the liver. The findings suggested that EDR might be a natural preparation possessing antioxidant and anti-inflammatory properties. Although this study provided some experimental evidences for the anti-inflammatory and antioxidant effects of EDR, the active components in the extracts are still unknown as well as the mechanisms of action. Therefore, it is necessary to isolate the active compositions from the extracts of DRF guided by the pharmacological studies. Further, the anti-inflammatory and antioxidant mechanisms of the extracts are encouraged by supplementing specific agonists or antagonists of inflammation- or oxidation-related signaling molecules such as NF-κB or NO to assay changes in the pharmacokinetics in the presence of EDR.

Qu et al. (2003a) investigated the anti-inflammatory effects of n-butanol extracts of DRF (NDR) by using capillary permeation and ear swelling in mice and foot edema in rats. 1 h post the last administrations, all the mice were injected with 2% evans blue via tail veins (0.1 ml/10 g). 10 min after the injections, the animals were intraperitoneally injected with 0.6% acetic acid (0.1 ml/10 g). Another 20 min later, the animals were sacrificed and the abdominal cavities were cut open and washed with 2 ml of NS for 3 times. 5～6 ml of peritoneal eluate was centrifuged at 1,000 rpm for 5 min. The supernatant was harvested and the concentration of evans blue was calculated by measuring OD value at 590 nm. The result showed that the concentrations of evans blue in the NDR-treated groups (8.2, 16.3, 32.5 g/kg) were lower than the NS group (63.41 ± 28.11, 52.21 ± 32.91, 50.517 ± 30.59 μg/ml vs. 136.61 ± 26.82 μg/ml, all *p* < 0.01), which indicated that NDR reduced acetic acid-induced increased capillary permeability. In xylene-induced ear swelling experiment, 1 h after the last administrations, xylene was applied on the right ear of each mice (0.1 ml/ear). The left ear was selected as a normal control. 60 min after the inflammations, the animals were sacrificed and the ears of the both two sides were taken off by using a 6-mm hole puncher. The difference in the ears of the both two sides was used to assess the ear swelling. The result demonstrated that compared with the NS, indomethacin (20 mg/kg, i.g.), NDR (8.2 and 32.5 g/kg, i.g.) significantly reduced xylene-induced ear swelling (10.08 ± 5.37, 17.75 ± 7.11, 20.67 ± 9.43 mg vs. 30.33 ± 11.19 mg, *p* < 0.01, *p* < 0.01, *p* < 0.05, respectively), with the inhibition rates of 66.77%, 41.48% and 31.85%, respectively. In carrageenan-induced paw edema experiment, 30 min after the last administrations, 1% carrageenan was injected into the right behind paw of each rat (0.1 ml/rat). After that, the paw thickness was measured by using a vernier caliper at specific time points within 6 h. The result revealed that the low-dose NDR (8.2 g/kg, i.g.) inhibited the carrageenan-induced paw edema 6 h post the inflammation, and the high-dose NDR (32.5 g/kg) alleviated the paw edema 2 and 3 h after the inflammation. These findings suggested that NDR exhibited anti-inflammatory effects in acute inflammation-related animal models. However, some issues still exist in this study, including 1) genders of the mice and rats. Since estrogen exhibits anti-inflammatory effect (Cheng et al., 2019), it is more reasonable to use only male animals for the evaluation of anti-inflammatory activity. 2) the same doses of NDR used in mice and rats. Due to varied body surface areas of animal species, the effective doses of a same drug used in mouse and rat are thought to be different.

Zeng et al. (2005) assessed the anti-inflammatory effects of petroleum ether extracts of DRF (PER) by establishing ear swelling and paw edema models in mice. The result showed that the middle-dose PER (2.5 g/kg) significantly inhibited xylene-induced ear swelling compared with the NS (17.20 ± 5.95 mg vs. 25.40 ± 7.93 mg, *p* < 0.05). In addition, the results of egg albumen-induced paw edema experiment found that PER (1.25, 2.5 and 5 g/kg, i.g.) remarkably inhibited egg albumen-induced paw edema at some time points. These findings suggested that PER markedly suppressed acute inflammation reactions induced by chemicals or extrinsic proteins. Similar to the study of Qu et al. (2003a), the choice for the gender of the mice was also inappropriate. Also, it is a preliminary study on the anti-inflammatory effects of the extracts from DRF. So, chemical studies on active components are needed as well as research on mechanisms of action of the extracts from DRF.

### Antipyretic Effects

The antipyretic effects of DRF are linked to its heat-clearing and detoxification (The minister of health of logistics department of Kunming military command, 1970; Guangxi health administration, 1970; Fujian institute of medicine, 1970). An imbalance of heat production and heat dissipation results in dysfunction of body temperature center, causing occurrence of fever. In the development of fever, the roles of PGE2 and COX-2 are conspicuous (Pakai et al., 2018).

Qu et al. (2003b) evaluated antipyretic effects of NDR by subcutaneous injections of 10% peptone on the backs in rats (1 g/kg, i.g.). All the rats received the injections of 10% peptone on the backs at the third day. Subsequently, all the animals were treated once as before. After that, the temperature of each rat was detected every 1 h and lasted 4 h continuously. Compared with the NS, low-dose NDR (8.2 g/kg, i.g.) significantly reduced increased temperature 2 h post the injections. However, middle-dose NDR (16.3 g/kg, i.g.) had no significant inhibitory effect on peptone-induced pyrogenicity. High-dose NDR (32.5 g/kg, i.g.) markedly reduced increased temperature 1, 3 and 4 h after the injections, respectively. These findings suggested that NDR displayed significant antipyretic effects in peptone-induced pyrogenicity rat model. However, it is necessary to discuss the dose-efficacy of NDR in the antipyretics experiment. Additionally, it is encouraged to determine the key molecules like PGE2 and COX-2 that are closely related to the antipyretic mechanism.

### Analgesic Effects

The anti-inflammatory effects of DRF contribute to its analgesic effects (The minister of health of logistics department of Kunming military command, 1970). Commonly, the inflammatory cytokines such as TNF-α, IL-6, and others contribute to inflammation-induced pain.

Qu et al. (2003a) evaluated the analgesic effects of NDR by establishing 0.6% acetic acid-induced abdominal writhing model. 1 h after the last administrations, the animals were intraperitoneally injected with 0.6% acetic acid (0.2 ml/mouse). Recorded the number of writhing in each mouse within 15 min. The result showed that the numbers of writhing in rotundine and middle-dose NDR (16.3 g/kg, i.g.) groups were, respectively, lower than the NS group. It suggested that NDR alleviated acetic acid-induced peripheral inflammatory pain.

Similarly, Zeng et al. (2005) evaluated the analgesic effects of PER by using acid-induced abdominal writhing in mice described as Qu et al. (2003a). The result demonstrated that middle-dose PER (2.5 g/kg, i.g.) reduced the number of acetic acid-induced writhing and displayed significant antinociceptive effects.

These two studies preliminarily evaluated the analgesic effects of the extracts from DRF *in vivo*. However, it is necessary to clarify the mechanisms linking to the inflammatory cytokines including TNF-α, IL-6, etc. for the antinociceptive effects of these extracts. In addition, it needs to clarify the details for dose-efficacy of NDR and PER in the analgesics experiments.

### Antibacterial Effects

The antibacterial effects of DRF contribute to its heat-clearing and detoxification (Editorial board of Chinese materia medica and State administration of traditional Chinese medicine, 2005).

An *in vitro* study assessed antibacterial effects of NDR (Qu et al., 2003a) by using disc-agar diffusion. The results demonstrated that MIC and MBC of NDR against *Staphylococcus aureus* were 0.102 and 0.407 mg crude drug/ml, respectively; the MIC and the MBC against beta-hemolytic *streptococcus* were 0.102 and 0.813 mg crude drug/ml, respectively. The antibacterial susceptibility test showed that NDR had strong antibacterial effects on Gram-positive pathogenic cocci such as *Staphylococcus aureus* and beta-*hemolytic Streptococcus*, while not on Gram-negative bacilli including *Escherichia coli, Salmonella typhi, Proteus vulgaris*, and *Shigella Castellani*.

The essential oils from DRF were used to evaluate their antibacterial activities also by using the disc-agar diffusion (Wu et al., 2009). The results revealed that the essential oils inhibited all microorganisms tested including Gram-positive and -negative pathogenic bacteria. In particularly, the essential oils exhibited the strongest bactericidal effect on escherichia *coli* with the lowest MIC (0.8 μg/ml) and the lowest MBC (1.5 μg/ml). 6-(methylamino) phenanthren-3-ol belonging to phenolic compound is the highest content in the essential oils of DRF. Moreover, it possesses antimicrobial activity. Thus, the antimicrobial nature of the essential oils from DRF is mainly thought to be related to this component.

Although these studies evaluated the anti-bacterial properties of the extracts from DRF *in vitro*, it is encouraged to clarify the effective components and their SARs. Further, bacteria-induced infection models in animals are needed to understand the efficacies and mechanisms of the extracts or essential oils from DRF *in vivo* more comprehensively.

### Anti-tumor Effects

Qiu et al. (2016) evaluated anti-tumor activities of 20 compounds (5～24) in two human hepatocellular carcinoma cell lines QGY-7701 and SMMC-7721 and one human lung cancer cell line A549 by the MTT method. The results demonstrated that the compounds (7, 8, 14) exhibited strong anti-tumor effects in the QGY-7701 cells with IC_50_ values of 16.08, 10.79 and 8.84 μmol/L, respectively. In the SMMC-7721 cells, the IC_50_ values of the compounds (6, 8, 14) were respectively 19.89, 12.09, and 6.66 μmol/L. The compounds (6, 8, 10, 15, 24) displayed anti-tumor activities in the A549 cells with the IC50 values of 17.85, 8.21, 16.61, 3.42, and 16.92 μmol/L, respectively. Among these compounds, the compound (14) had the lowest IC50 values that were respectively lower than the positive control 5-FU in these three tumor cell lines, suggesting it had the most prominent anti-tumor activity *in vitro*. Further, the structure-activity relationships of these anti-tumor compounds were assayed. When AcO substituent was hydrolyzed into OH at position 10 of MTS derivatives, the cytotoxic activities were remarkably enhanced e.g., (16) vs. (6), (17) vs. (7), (18) vs. (8) except for compound (23). The findings suggested that the compound (14) might be a promising agent for the treatment of hepatocellular carcinoma and lung cancer in the future. Due to the relatively few compounds isolated from DRF, a more accurate conclusion on the structure–activity relationships for anti-tumor effects may be drawn from a larger number of MTS derivatives. Moreover, based on the previous findings, it is encouraged to investigate the efficacy of compound (14) in tumor-related animal models as well as the mechanisms of action.

Song et al. (2015) isolated three new resin glycosides dichondrins A–C (110～112) and three known resin glycosides (113～115) from DRF. Their multidrug resistance -reversal (MDR) effects were evaluated by the MTT method in oral cancer KB/VCR cells. The results showed that the isolated compounds (110～115) from DRF had no significant cytotoxicity at a concentration of 25 μmol/L with less than 50% of inhibition ratios. Interestingly, these compounds markedly enhanced the cytotoxicity of vincristine by 1.03～1.78-fold at this concentration. In addition, the two deacylated resin glycosides (112, 115) were more active than the two acylated resin glycosides (113, 114), suggesting their MDR reversal activities were affected by the minor variations in the acylation pattern of oligosaccharide core.

Wang and Xuan (2015) evaluated anti-tumor activities of a new highly oxygenated acyclic sesquiterpenoid (116) and its glucoside (117), and a new pinane monoterpene (118) isolated from hydrophilic extract of DRF in A549 and NCIH661 cell lines by sulforhodamine B (SRB) assay. Doxorubicin was selected as a positive control. However, all of these compounds (116～118) showed no significant cytotoxic activity with IC_50_ values all larger than 20 μmol/L.

### Hepatoprotective Effects

The hepatoprotective effects of DRF are based on its anti-HBV and anti-inflammatory properties (The minister of health of logistics department of Kunming military command, 1970; Guangxi health administration, 1970; Fujian institute of medicine, 1970).

Qu et al. (2003c) evaluated the hepatoprotective effects of NDR in D-GlaN-, TAA-, and ANIT-induced liver injury mouse models, respectively. 1 h after the last administrations, D-GlaN (800 mg/kg), TAA (50 mg/kg) or ANIT (100 mg/kg) was intraperitoneally injected in each animal in these groups except the negative group, respectively. 16, 20 or 30 h after the injections, the peripheral blood was collected and the sera were harvested for measurements of related parameters including ALT, AST, TG and Tbil. In the D-GlaN-induced liver injury model, NDR (8.2, 16.3 g/kg, i.g.) significantly reduced increased ALT and AST activities (ALT: 268.71 ± 181.62, 239.51 ± 204.47 U/l vs. 520.71 ± 195.11 U/l, *p* < 0.01, *p* < 0.01; 396.11 ± 253.27, 426.21 ± 197.27 U/l vs. 623.01 ± 165.91 U/l, *p* < 0.05, *p* < 0.05). In addition, the TG level in the middle-dose DRE-treated group (16.3 g/kg, i.g.) was lower than that in the D-GlaN-induced liver injury model group (0.74 ± 0.27 mmol/L vs. 1.95 ± 1.61 mmol/L, *p* < 0.05). NDR (8.2, 16.3 and 25.6 g/kg, i.g.) markedly reduced TAA-induced increased ALT level compared with the model control (330.61 ± 160.81, 397.51 ± 101.74, 401.21 ± 93.09 U/l vs. 518.12 ± 28.98 U/l, all *p* < 0.01). Further, Tbil level and ALT and AST activities were respectively lower in the middle-dose NDR (16.3 g/kg, i.g.) group than those in the ANIT-induced liver injury group (Tbil: 26.65 ± 11.03 μmol/L vs. 42.74 ± 16.21 μmol/L, *p* < 0.05; ALT: 262.41 ± 191.51 U/l vs. 398.31 ± 100.34 U/l, *p* < 0.05; AST: 433.12 ± 192.12 U/l vs. 626.71 ± 152.12 U/l, *p* < 0.05).

The hepatoprotective effect of NDR was also evaluated in a CCl_4_-induced liver injury mouse model (Qu et al., 2003d). 1 h after the last administrations, 0.1% CCl_4_ peanut oil solution (10 ml/kg) was intraperitoneally injected in each mouse except in the negative control group. 16 h after the injections, the peripheral blood was collected by eyeball removal and the sera were harvested after centrifuged at 3,000 rpm for 10 min. The activities of the serum ALT and AST were measured by the Reitman-Frankel method. The pathological examination was performed by using H&E staining. The results showed that NDR (8.2 and 16.3 g/kg) significantly reduced the serum AST not ALT activity compared with the model. The pathology results found obvious and extensive steatosis in the liver cells in the model group, accompanied by focal necrosis, infiltration of inflammatory cells, expansion of central vein, and loss of liver glycogen. Although these pathological changes existed in the DRE-treated group, the lesions were less than the model group.

These two studies evaluated the hepatoprotective effect of NDR from DRF in various liver injury models induced by different chemicals, which provided some experimental evidences for NDR in the treatment of liver injury-related diseases. However, the efficacies of various doses of NDR in the different models are varied and it needs clarifications. Further, described as before, it is necessary to explain the dose-efficacy relationship of NDR in these liver injury models.

### Cholagogic Effects

Anti-inflammatory effects of DRF contribute to its cholagogic effects (The minister of health of logistics department of Kunming military command, 1970; Guangxi health administration, 1970; Fujian institute of medicine, 1970).

Qu et al. (2003b) evaluated the cholagogic effects of NDR by using ligations of bile ducts in rats. The animals were anesthetized by intraperitoneal injections of 25% ethyl urethane (1 g/kg). The 40 rats were randomly divided into a NS group, a dehydrocholic acid (positive control) and low-, middle- and high-dose NDR groups (n = 8). Cut and opened the abdominal cavity with a 3 cm incision. Then ligated and fixed the bile duct. A V_0_-shape incision was made in the direction toward liver, and a capillary drainage tuber (diameter 1～2 mm) was inserted. Collected the bile within 30 min after the operation, and then respectively injected NS (10 ml/kg), NDR (8.2, 16.3, and 32.5 g/kg) and dehydrocholic acid tablet suspension in the duodenum (2 g/kg) into the tube. The bile was collected every 30 min after the injection within 120 min and compared the secretion of bile among groups at each time spot. The results showed that after the injections the bile flows in the NDR groups (8.2, 16.3, and 32.5 g/kg) were respectively higher than the NS group, suggesting NDR had a strong choleretic effect. Described as before, it is also necessary to investigate the cholagogic mechanisms for this extract from DRF.

### Immunomodulatory Effects

Qu et al. (2003b) evaluated immunomodulatory effects of NDR by weighting immune organ’s weights and calculating their indices, measuring carbon clearance and serum hemolysin level in mice.

The morning after the last administrations, the animals were sacrificed by decapitation and thymus and spleen were collected for weighting. Then the thymus and spleen indices were calculated. The results indicated that NDR (8.2 and 16.3 g/kg, i.g.) not only significantly increased the weights of the thymus and spleen, but also elevated the thymus and spleen indices compared with the NS control.

In addition, the animals were treated with NDR (8.2, 16.3 and 32.5 g/kg, i.g.) for 6 days and once a day. 1 h after the last administrations, 20% indian ink (diluted by NS, v/v) was injected into each mouse via tail vein (0.1 ml/10 g). 2 and 10 min after the injections, spiked ocular venous plexus and collected 25 μL of peripheral blood by using a sharp-mouthed pipettes preliminarily moistened with heparin. Added 2 ml of sodium carbonate solution in the blood sample and mixed well. The OD value of the mixed solution was measured at 600 nm. The phagocytic rate K was calculated according to the formula as follows. K = (logOD_1_-logOD_2_)/(t_2_-t_1_). Finally, weighed the livers and spleens to correct the K values. The corrected K is calculated as × body weight/(liver weight + spleen weight). The results revealed that DRE (8.2, 16.3, 32.5 g/kg, i.g.) remarkably increased the K value and the corrected K value of carbon clearance, suggesting that this preparation enhanced phagocytic function of mononuclear macrophages.

Further, a chicken red blood cells-induced immune model was established to detect level of serum hemolysin. At the ninth day, the peripheral blood was collected by enucleation of eyeball. Centrifuged at 3,000 rpm for 10 min and supernatant was harvested. Then the supernatant was diluted by a 100-fold. Subsequently, added 0.5 ml of 5% chicken red blood cells and 0.5 ml of 10% complement (guinea pig serum) in 1 ml of the diluted supernatant. Mixed and incubated in a water bath at 37°C for 30 min. Stopped the reaction at 0°C. Next, the reaction solution was centrifuged at 3,000 rpm for another 10 min. Finally, the OD value of the supernatant was determined at 540 nm. The results showed that NDR (16.3 and 32.5 g/kg, i.g.) markedly elevated the level of serum hemolysin compared with the NS control, suggesting NDR effectively enhanced specific immunity (humoral immunity) function of the mice induced by chicken red blood cells.

In summary, NDR significantly enhanced cellular immunity and humoral immunity. According to the previous experimental results, NDR might be useful in enhancing immune function of body to potentially treat some diseases associated with low immune, such as influenza, aplastic anemia, tumors, and others. However, some issues also occurred, including 1) the durations of NDR were varied from 6 to 10 days in these three experiments evaluating immunomodulatory effects; 2) the dose-efficacy relationship of the NDR also needed clarifications as well as the mechanisms; 3) the positive controls in all the three experiments were absent and levamisole might be an appropriate choice.

## Toxicological Studies on Extracts and Compounds From DRF

Except bentysrepinine, few studies have been conducted on toxicities of the extracts or compounds from DRF.

*In vitro*, the inhibitory effect of bentysrepinine on cell proliferation of HepG2 was evaluated by using the MTT method. Fluorescent-activated cell sorting (FACS) was used to detect mitochondrial membrane potential (MMP) and reactive oxygen species (ROS) content of the cells. In addition, level of lactic acid released from the HepG2 cells was determined by colorimetry. Activities of mitochondrial respiratory chain complex enzymes I, II, III and IV were assayed by coomassie blue staining (Feng et al., 2017). The results revealed that IC_50_ of bentysrepinine was 359 μmol/L in the HepG2 cells. Compared with the control, bentysrepinine (400 μmol/L or 196 mg/L) significantly reduced the MMP and mitochondrial respiratory complex enzymes I, II and III activities (Table 7), and remarkably increased the ROS content and the level of lactic acid, suggesting a significant mitochondrial toxicity. Compared with the positive controls lamivudine and adefovir dipivoxil, bentysrepinine had no significant effects on these indices above at a same concentration of 100 μmol/L. These findings showed that safety range of the mitochondrial toxicity of bentysrepinine was relatively wide, which might provide some guides for dosage design of clinical trial and clinical medication.

**TABLE 7:** Effect of bentysrepinine on activity of mitochondrial respiratory chain complex enzymes of HepG2 cells (±s, n = 3).

Group	Concentration/μmol/l	COX I	COX II	COX III	COX IV
Control	–	31.42 ± 1.52	50.75 ± 1.78	15.86 ± 1.06	4.29 ± 2.48
Bentysrepinine	100	30.19 ± 2.01	48.16 ± 1.68	15.64 ± 1.04	3.59 ± 1.02
	200	28.50 ± 1.85	49.23 ± 1.70	15.60 ± 1.27	3.08 ± 1.54
	400	19.73 ± 1.30^a^	29.77 ± 1.32^a^	13.63 ± 0.89^b^	2.21 ± 1.04
Lamivudine	100	25.40 ± 1.18^a^	45.40 ± 2.05^b^	15.54 ± 1.03	2.90 ± 1.35
Adefovir dipivoxil	100	20.00 ± 1.56^a^	50.95 ± 1.86	14.56 ± 1.05	2.86 ± 1.43

^a^ p < 0.01 vs. Control. Unit of enzyme activity: nmol cytochrome C/min mg.

^b^ p < 0.05.

*In vivo*, 71 pregnant SD rats were continuously administrated with various doses of bentysrepinine (50, 175 and 620 mg/kg for 24, 22, and 25 animals, i.g., respectively) from 6th to 15th day of the pregnancy and once a day (Wen et al., 2018). Meanwhile, 20 pregnant SD rats in the vehicle group were given 0.5% CMC-Na. The results indicated that compared with the vehicle control group, the body weight, food intake, and body weight growth of the pregnant rats in bentysrepinine-treated group (175 and 620 mg/kg) significantly reduced during the administration period. Bentysrepinine (50, 175, and 620 mg/kg, i.g.) had no significant effect on fertility of the pregnant rats. The fetal body weight was relative lower in bentysrepinine-treated group at the dose of 620 mg/kg. Observed dysplasia including reduced body weight and increased wavy rib after treated with bentysrepinine at 620 mg/kg. Some systemic toxicities occurred in the pregnant rats treated with bentysrepinine at 175 and 620 mg/kg. Therefore, 50 mg/kg (30 times of planned dose in clinic) was a non-toxic dose for bentysrepinine used in the pregnant rats. Therefore, bentysrepinine had some certain toxicity to the embryo development at 620 mg/kg, while 175 mg/kg was a non-toxic reactive dose.

A Phase I clinical trial has studied tolerance and pharmacokinetics of bentysrepinine tablet by a randomized and double-blind method (Liang et al., 2020). The results showed that the t_max_ and t_1/2_ were respectively 1.02 ± 0.13 h and 3.08 ± 0.09 h after taking different doses of the bentysrepinine tablet for several times. The t_max_ and t_1/2_ of one metabolite N-benzoyl-O-(dimethylaminoethyl)-l-tyrosine (M8) were, respectively, 3.05 ± 0.07 h and 9.56 ± 0.67 h. Correspondingly, the t_max_ and t_1/2_ were, respectively, 4.11 ± 0.15 h and 6.17 ± 0.21 h for another metabolite N-benzoyl-l-tyrosine-O-(N-oxidation -dimethylaminoethyl)-l-tyrosine (M9). After taking 300 mg of bentysrepinine, there were no significant differences in C_max_, C_min_, C_avg_, AUC_0-t_, AUC_0-∞_ and AI between the two groups. For M8 and M9, AUC_0-t_ and AUC_0-∞_ in the multiple doses were higher than those in the single dose. For M9, the C_max_ was significantly increased in the multiple doses compared with the single dose; After taking 600 mg of bentysrepinine, the C_max_ in the multiple doses was higher than that in the single dose. No significant differences were found in C_min_, C_avg_, AUC_0∼t_, AUC_0-∞_ and AI between the two groups; For M8 and M9, C_max_ and AUC_0∼t_ were higher in the multiple doses than the single dose. Compared with the single dose, the AUC_0-∞_ of M9 was markedly elevated in the multiple doses. The multiple doses of 600 mg remarkably increased the C_max_, but had no significant influence on the exposure of AUC_0-∞_. Along with the increases in the C_max_ values of M8 and M9, the exposures of AUC_0-∞_ increased (Table 8). After oral administrations of the bentysrepinine tablet in the healthy volunteers for several times, a case of slight throat discomfort occurred in one volunteer at the dose of 600 mg, which was finally thought to be unrelated to the drug. No volunteer withdrew from the trial for the adverse reactions. The oral dose of 600 mg of the bentysrepinine tablet showed a high safety with no significant accumulation effect. It suggested that 600 mg might be an appropriate daily dose for bentysrepinine to exert its antiviral effect in clinic.

**TABLE 8:** Pharmacokinetic parameters in single or multiple doses of bentysrepinine ant its metabolites in healthy subjects.

Dose	C_max_ (ng/ml)	C_min_ (ng/ml)	C_avg_ (ng/ml)	AUC_0-t_	AUC_0-∞_	AI
Bentysrepinine 300 mg (n = 24)						
Single	521.21 ± 162.72	5.03 ± 1.53	60.06 ± 8.42	1,314.61 ± 209.52	1,334.78 ± 205.35	0.98 ± 0.21
Multiple	607.13 ± 120.18	5.12 ± 1.49	60.11 ± 8.31	1,436.32 ± 211.23	1,458.78 ± 227.83	1.08 ± 0.25
t	2.523	1.528	1.957	2.315	2.204	1.912
P	0.332	0.581	0.504	0.385	0.397	0.521
Bentysrepinine 600 mg (n = 24)						
Single	1,369.13 ± 431.25	5.71 ± 2.27	151.32 ± 51.22	3,765.27 ± 1,206.33	3,812.71 ± 1,215.24	0.96 ± 0.09
Multiple	1769.41 ± 636.33	5.89 ± 2.41	159.10 ± 50.64	3,793.41 ± 1,218.50	3,831.89 ± 1,207.49	1.01 ± 0.02
t	4.135	2.561	1.422	1.016	1.025	2.694
P	0.036	0.269	0.655	0.851	0.812	0.126
M8 300 mg (n = 24)						
Single	1,687.16 ± 364.46	33.07 ± 2.27	504.16 ± 81.25	11,514.99 ± 2,156.86	11,610.96 ± 2,152.33	1.18 ± 0.13
Multiple	1866.28 ± 259.55	33.85 ± 11.44	510.41 ± 89.70	12,762.02 ± 2,335.99	12,961.68 ± 2,383.29	1.27 ± 0.20
t	1.877	2.663	2.715	5.153	5.249	2.155
P	0.524	0.158	0.089	0.022	0.021	0.412
M8 600 mg (n = 24)						
Single	3,291.45 ± 776.72	81.24 ± 20.38	1,006.31 ± 198.29	23,158.84 ± 4,050.39	23,348.32 ± 4,055.41	1.11 ± 0.21
Multiple	3,763.51 ± 939.73	85.56 ± 23.63	1,018.96 ± 205.47	25,475.03 ± 5,021.00	25,766.88 ± 5,189.90	1.19 ± 0.23
t	4.357	2.917	2.592	5.631	2.416	1.633
P	0.031	0.073	0.245	0.019	0.348	0.549
M9 300 mg (n = 24)						
Single	186.91 ± 47.64	4.68 ± 1.43	71.24 ± 15.98	1,639.48 ± 345.26	1,650.82 ± 346.27	1.09 ± 0.23
Multiple	230.65 ± 60.48	4.91 ± 1.59	76.66 ± 17.20	1908.26 ± 417.01	1925.91 ± 414.66	1.14 ± 0.22
t	3.856	1.314	1.369	4.372	4.679	1.489
P	0.041	0.717	0.696	0.029	0.025	0.617
M9 600 mg (n = 24)						
Single	485.53 ± 215.66	13.61 ± 3.66	187.39 ± 75.66	3,919.60 ± 1,282.19	3,938.27 ± 1,282.76	1.01 ± 0.04
Multiple	595.31 ± 290.18	13.97 ± 3.89	191.38 ± 78.08	4,745.58 ± 1894.35	4,764.74 ± 1897.03	1.09 ± 0.09
t	3.719	1.945	2.014	3.814	3.926	3.126
P	0.045	0.516	0.487	0.043	0.039	0.067
						

Liu et al. (2020) also evaluated safety, tolerability, and pharmacokinetics of bentysrepinine by using a single oral dose (50～900 mg), multiple doses (300 mg and 600 mg), and a randomized dose (600 mg) considering food-effect in 94 healthy subjects by using two randomized, double-blind, placebo-controlled trials. The results found mild and reversible adverse events in single and multiple oral doses between 50 and 900 mg. The most common adverse effects were increased ALT and AST, followed by nausea, elevated urine leukocytes, urine red blood cells, and others. T_max_ and t_1/2_ of bentysrepinine were respectively 1～2 h and 1～3 h, suggesting rapid absorption, metabolism, and elimination of this drug. Thus, during the dose escalation study the maximum tolerated dose (MTD) was not detected. The AUC and the C_max_ of bentysrepinine were elevated in a dose-dependent manner in the single dose study (50～900 mg). No accumulation was found after the oral administration of 300 mg and 600 mg for several times in the multiple dose study, which was consistent with the study of Liang et al. (2020). Further, this study revealed that food intake had significantly increased the absorption of bentysrepinine. In addition, no differences were found in the results between females and males (Table 9～12).

**TABLE 9:** Pharmacokinetic parameters of bentysrepinine in a single oral dose (Study 01) and a randomised, open, crossover food-effect study (Study 03).

Dose (mg)	C_max_ (ng/ml)	T_max_ (h)	t_1/2_ (h)	AUC_0-t_ (ng·h/ml)	AUC_0-∞_ (ng·h/ml)	CL/F (l/h)	V/F (L)
Study 01							
50	54.2 (24.5)	1.50 (0.50–3.00)	1.7 (1.1)	106.1 (26.5)	110.2 (25.1)	481.1 (148.6)	1,373.0 (1,441.8)
100	127.3 (43.9)	2.30 (0.50–5.00)	1.7 (1.1)	349.0 (83.4)	354.1 (84.3)	299.7 (85.8)	726.2 (269.8)
200	356.7 (162.3)	0.90 (0.50–2.50)	2.1 (0.7)	864.2 (209.3)	870.9 (208.9)	241.1 (55.1)	735.3 (344.9)
400	921.3 (267.6)	1.50 (0.50–2.00)	4.2 (3.2)	2,232.7 (494.7)	2,246.0 (494.4)	185.1 (37.7)	1,097 (764.5)
600	1,395.5 (766.1)	2.00 (0.75–5.00)	3.5 (1.4)	4,002.9 (1,089.4)	4,056.2 (1,055.2)	157.6 (43.6)	771.7 (301.5)
900	1712.9 (602.5)	1.50 (0.75–4.00)	3.2 (1.4)	5,115.4 (1,281.1)	5,151.4 (1,276.8)	182.8 (38.4)	854.0 (472.9)
Study 03							
600 (fasted)	1,126.4 (391.1)	1.50 (0.66–3.00)	2.9 (2.0)	3,312.6 (1,271.5)	3,330.4 (1,271.8)	208.4 (88.3)	845.8 (556.1)
600 (fed)	1,258.6 (613.2)	1.75 (1.00–4.00)	2.6 (1.9)	3,635.8 (923.8)	3,652.6 (920.0)	174.3 (46.0)	690.0 (638.5)

The data are expressed as mean (standard deviation, SD).

**TABLE 10:** Pharmacokinetic parameters of bentysrepinine in male and female subjects in Study 01.

Dose (mg)	Gender	C_max_ (ng/ml)	T_max_ (h)	t_1/2_ (h)	AUC_0-t_ (ng·h/ml)
50	Male	51.5 (14.3)	2.25 (0.50–3.00)	1.3 (0.2)	110.2 (7.4)
100		108.8 (45.1)	3.25 (0.50–5.00)	1.9 (0.2)	312.7 (90.8)
200		281.1 (35.0)	0.88 (0.50–1.00)	2.5 (0.7)	799.8 (219.6)
400		806.0 (83.9)	0.75 (0.50–2.00)	3.5 (1.6)	2,103.4 (246.8)
600		1,618.6 (1,031.7)	1.13 (0.75–5.00)	2.8 (1.5)	3,582.4 (1,089.4)
900		1,387.0 (270.2)	1.75 (0.75–4.00)	3.3 (1.6)	4,555.1 (518.5)
50	Female	59.7 (48.1)	1.00 (1.00–1.00)	2.5 (2.0)	97.8 (56.1)
100		145.7 (39.5)	1.50 (0.50–3.00)	1.4 (0.1)	385.2 (66.9)
200		432.4 (212.0)	1.63 (0.50–2.50)	1.7 (0.4)	928.6 (207.3)
400		990.5 (325.4)	1.50 (0.50–2.00)	4.7 (4.1)	2,310.3 (614.7)
600		1,172.5 (415.3)	2.50 (2.00–4.00)	4.2 (1.1)	4,423.4 (1,087.7)
900		2038.8 (700.6)	1.50 (1.50–2.00)	3.1 (1.3)	5,675.7 (1,650.4)

The data are expressed as mean (SD).

**TABLE 11:** Pharmacokinetic parameters of bentysrepinine, M8 and M9 in a multiple doses study (Study 02).

Analyte	Dose (mg)	Day	C_max_ (ng/ml)	C_min_ (ng/ml)	T_max_ (h)	t_1/2_ (h)	AUC_0-t_ (ng·h/ml)	CL/F (l/h)	V/F (L)	R1	R2
Bentysrepinine	300	1	521.2 (162.7)	—	0.88 (0.50–5.00)	1.6 (0.4)	1,314.6 (209.5)	229.4 (34.0)	535.3 (185.7)	—	—
	300	9	607.3 (120.8)	5.0 (1.5)	1.00 (0.50–2.00)	3.6 (5.9)	1,436.3 (211.2)	212.0 (31.1)	535.3 (185.7)	1.10 (0.20)	1.10 (0.20)
	600	1	1,368.7 (431.4)	—	1.50 (0.50–2.50)	3.7 (2.4)	3,507.8 (765.5)	175.5 (32.2)	883.5 (577.3)	—	—
	600	9	1769.4 (636.1)	5.9 (2.4)	1.25 (0.50–2.50)	2.8 (1.4)	3,793.4 (1,218.5)	169.6 (45.2)	722.2 (494.7)	1.33 (0.37)	1.09 (0.26)
M8	300	1	1,687.5 (364.2)	—	4.00 (2.00–6.00)	6.2 (3.9)	11,515.0 (2,156.9)	26.6 (4.7)	239.9 (166.4)	—	—
	300	9	1866.1 (259.1)	33.9 (11.4)	3.00 (2.50–6.00)	10.3 (4.4)	12,762.0 (2,336.0)	25.2 (4.4)	379.0 (194.8)	1.13 (0.19)	1.10 (0.14)
	600	1	3,290.8 (776.2)	—	3.50 (2.50–4.00)	8.2 (2.5)	23,158.8 (4,050.4)	26.4 (4.4)	312.0 (110.9)	—	—
	600	9	3,763.3 (938.8)	85.6 (23.6)	3.00 (2.00–4.00)	8.6 (4.8)	25,475.0 (5,021.0)	25.5 (5.1)	308.3 (149.3)	1.15 (0.17)	1.10 (0.10)
M9	300	1	186.9 (47.6)	—	5.50 (4.00–6.00)	4.3 (1.2)	1,639.5 (345.3)	192.2 (57.5)	1,161.4 (368.1)	—	—
	300	9	230.6 (60.4)	4.9 (1.6)	4.00 (4.00–6.00)	7.1 (4.8)	1908.3 (417.0)	172.3 (47.5)	1826.4 (1,368.1)	1.25 (0.21)	1.15 (0.12)
	600	1	485.6 (215.6)	—	4.00 (4.00–6.00)	5.9 (1.9)	3,919.6 (1,282.2)	169.6 (60.3)	1,430.3 (626.5)	—	—
	600	9	595.3 (290.2)	14.0 (3.9)	4.00 (3.00–5.00)	6.2 (2.3)	4,745.6 (1894.3)	148.4 (52.5)	1,329.4 (546.4)	1.24 (0.22)	1.22 (0.21)

The data are expressed as mean (SD). R1 = C_max ss_/C_max_; R2 = AUC _0-τ ss_/AUC _0-τ_.

**TABLE 12:** Pharmacokinetic parameters of bentysrepinine, M8 and M9 in a multiple doses study (Study 02).

Analyte	Urine		Feces	
	Cumulative Ae (μmol/L)	Cumulative %Fe (%)	Cumulative Ae (μmol/L)	Cumulative %Fe (%)
Bentysrepinine	24.4 (7.9)	3.0 (1.0)	37.4 (32.7)	4.6 (4.0)
M8	222.6 (44.6)	27.2 (5.5)	105.1 (50.5)	12.9 (6.2)
M9	20.2 (7.4)	2.1 (1.2)	—	—

The data are expressed as mean (SD). Ae: amount of drug excreted in urine or feces, %Fe: % of drug excreted in urine or feces.

In summary, these systemic studies above indicated that bentysrepinine had a wide range of safety to exert its anti-HBV activity. However, it is far from enough to comprehensively understand the toxicities of extracts or compounds from this plant except bentysrepinine. The toxicological effects of the extracts or compounds from DRF on central nervous system, cardiovascular system, respiratory system, urinary system, gastrointestinal system etc. should also be taken into consideration for safety evaluation in the future study.

## Modern Uses

Currently, DRF has been made into some preparations including Jinma gantai granules, Ganlexin capsule, Handanbituo granules, and Shangtongke spirit combined with other TCMs to treat hepatitis, liver fibrosis, cirrhosis (Cheng et al., 1996; Liu et al., 1997), contusions and strains (Qin et al., 2019) (Table 13), which is a prolongation of the traditional uses of this plant. However, the preparations containing DRF listed on market in China are few for the lacks of understanding in phytochemistry, pharmacology, toxicology, incompatibility with other TCMs, and others of this plant. Moreover, no single extract or compound from DRF has been listed in the market and applied to treat diseases in clinic until now. Thus, it is necessary to study and explore the extracts or compounds from DRF to treat diseases based on related studies above.

**TABLE 13:** The modern uses of DRF in China.

Name	Main compositions	Modern uses	Usage
Jinma gantai granules	DRF 288.2 g, Berchemia lineata (L.) DC. 153.3 g, verbena officinalis L. 115 g, stephania tetrandra S. Moore 191.6 g, Thlaspi arvense L. 115 g, Epimedium brevicornu maxim. 153.3 g, Astragalus mongholicus Bunge 153.3 g, Paeonia anomala subsp. Veitchii (Lynch) D.Y.Hong and K.Y.Pan 153.3 g, salvia miltiorrhiza Bunge 230 g, sucrose1048 g or dextrin 105g, 2% hydroxypropyl methylcellulose ethanol solution 80 ml (for 1000 g)	Curing acute and chronic hepatitis	Take orally 3 times a day (10 g once)
Ganlexin capsule	Rumex obtusifolius L. 250 g, Artemisia capillaris Thunb. 200 g, swertia bimaculata (siebold and Zucc.) Hook.f. and Thomson ex C.B.Clarke 200 g, Curcuma aromatica salisb. 120 g, Dimocarpus longan Lour. 10 g, Gardenia jasminoides J.Ellis 200 g, Phellodendron chinense C.K.Schneid. 200 g, DRF 200 g	Curing acute and chronic hepatitis	Take orally 3 times a day (0.9 g once)
Handanbituo granules	Stephania tetrandra S. Moore 20 g, salvia miltiorrhiza Bunge 20 g, Paeonia anomala subsp. Veitchii (Lynch) D.Y.Hong and K.Y.Pan 20 g, Thlaspi arvense L. 15g, DRF 12 g, verbena officinalis L. 10 g	Curing fibrosis	Take orally 3 times a day (15 g once)
Shangtongke spirit	Curcuma longa L. 180 g, DRF 80g, selaginella moellendorffii Hieron. 60 g (for 1,000 ml)	Curing contusions and strains	Apply externally 2～4 times a day

The full taxonomic names of the species have been validated using www.theplantlist.org.

## Conclusions-Limitations and Solutions for Current Research on DRF

After reviewing the studies on the traditional uses, chemistry, pharmacology and applications of DRF, some challenges for future studies on this plant have become conspicuous.

Except phenylalanine dipeptide compounds including MTS and its derivatives, the phytochemistry research on DRF is lacking in other types of compounds. Further, most of chemical studies focus on lipid-soluble components, while systemic works on hydrophilic parts are few.

The SAR analyses of anti-HBV activities of MTS and its derivatives haven’t been clearly clarified yet. Limited data showed that anti-HBV activities were relatively lower in fluorine- or chlorine-substituted derivatives of MTS compounds (25～29) with 4-hydroxy substitution in ring A, while the compounds (30, 32, 33) showed enhanced anti-HBV effects when 4-phenolic hydroxyl was methylated in the ring A. It indicated that methylation of 4-hydroxy substitution in ring A is crucial for the anti-HBV activity. In derivatives containing veratric acid, preliminary SAR analysis showed that when substituent R3 was hydroxymethyl, the derivatives containing hydroxy or acetoxy on the para position of the B ring had no anti-HBV activity. Interestingly, the compounds (51, 52) showed good activities against HBV when the hydroxy was substituted by the alkylation in the B ring. Thus, hydroxy substituted by alkylation is also an efficient method to elevate the anti-HBV activity. In addition, in derivatives with aromatic heterocycles the SAR analysis showed when benzene ring C of MTS was replaced by a furan ring or the para position of the ring A had an alkoxyl side chain, both anti-HBV activity and SI were significantly improved. It suggested that replacement of a furan ring or an alkoxyl side chain was beneficial to enhance the anti-HBV activity of MTS and its derivatives. Although the analyses are present as above, it still needs further investigations on the SARs of these compounds to modify and synthesize compounds with good anti-HBV activities.

Most of the chemical studies of DRF focus on the whole plant of this plant. However, until now no study has conducted on fruits and flowers of DRF individually. Furthermore, until now no study has reported differences in a same component in this plant regarding main production areas and seasons.

Although pharmacological studies have been conducted in MTS and its derivatives, some issues are still obvious: 1) except bentysrepinine, most of the anti-HBV activity studies on MTS and its derivatives still remain at cell level; 2) few studies have systemically investigated mechanisms of action of compounds or extracts from DRF, as well as SARs of the compounds derived from this plant; 3) except bentysrepinine, systemic toxicity studies on extracts or compounds of DRF are rare; 4) except bentysrepinine, studies on ADME of isolated compounds or extracts from this plant are absent for lacks in pharmacokinetics studies *in vivo*; 5) quality controls including selections of experimental animal gender, positive control, administration dose and duration are inaccurate in some pharmacological studies on the extracts or compounds of DRF.

Nowadays, in clinic DRF has been widely used to treat HBV infections-relative diseases like hepatitis, liver fibrosis, and cirrhosis, which is a prolongation of its traditional uses. To efficiently utilize this plant to maintain the health of people, it is worth doing some more beneficial works. In view of this, we will adopt the measures follows to solve the issues described as above.

Firstly, for varied contents of characteristic substances for example MTS and umbelliferone in DRF regarding main production areas and seasons, it is necessary to establish quality standards for medicinal components from this plant. Further, good agricultural practice (GAP) bases are needed to cultivate DRF to ensure quality of preparations or drugs from this medicinal plant.

Secondly, chemical components in water-soluble parts are required measurements and analyses. The chemical studies are encouraged to analyze active components from fruits and flowers based on pharmacological tests. Moreover, it is necessary to clearly clarify SARs of these compounds from this plant.

Thirdly, except anti-HBV activity, it is significant to validate efficacy of the extracts or compounds from DRF in some other specific diseases as well as the mechanisms of action. For its cholagogic effects, cholecystitis may enter a prior therapeutic scope of this plant. For its anti-inflammatory effects and anti-bacterial effects, it may be therapeutic for treatment of bacterial sepsis. For preliminary anti-tumor activity of the compounds from DRF at cell level, it is encouraged to validate their anti-tumor efficacies as well as mechanisms in xenograft models or carcinoma models *in situ*.

Fourthly, except bentysrepinine, few toxicological studies have been conducted in extracts or compounds of DRF. Therefore, studies on acute toxicity, chronic toxicity, safety pharmacology, reproductive toxicity, genotoxicity, and others are needed to systemically evaluate toxicities of extracts or compounds from this plant comprehensively in various experimental animals.

Fifthly, except bentysrepinine, no study reported pharmacokinetic parameters such as T_1/2_, AUC, and bioavailability of the compounds or extracts of DRF in various experimental animals. Thus, pharmacokinetic research is encouraged to gain related parameters for referencing further clinic studies.

Sixthly, a phase II trial is ongoing to validate the anti-HBV efficacy of bentysrepinine in HBV patients. Other extracts or compounds from DRF also need random, double-blinded, multi-center clinical trials. Through the clinical trials, it will provide important references for clinical applications of this plant.

In summary, this review provided a comprehensive and critical analysis of chemistry, pharmacology, toxicology, traditional and modern applications of DRF. As an ethnomedicine mainly used by Dai and Miao nations in the southwestern China, DRF exerts pharmacological effects mainly anti-HBV activity emphasized in the traditional and modern uses. This review also raised limitations and solutions for the research and development of this plant. Besides anti-HBV properties of MTS and its derivatives, we also summarized and analyzed the importance of DRF, and provided some new research directions for this ethnomedicine.

## Glossary

A549: human lung cancer cell line
ABTS: 2, 2′-azino-bis(3-ethylbenzothiazoline-6-sulfonic acid)
ACP5: acid phosphatase 5
ADME: absorption, distribution, metabolism, excretion
AI: accumulation index
AIDS: acquired immune deficiency syndrome
ALT: alanine aminotransferase
ANIT: α-nephthyl isothiocyanate
AST: aspartate aminotransferase
ATCC: american type culture collection
ATF4: activating transcription factor 4
AUC: area under the concentration-time curve
AUC_0-t_: area under the concentration-time curve form time zero to the time of last quantifiable concentration
AUC_0-∞_: AUC from time zero to infinity
Bcl-2: B-cell CLL/lymphoma 2
Carr: Carrageenan
CAT: Catalase
C_avg_: average concentration
CCl_4_: Carbon tetrachloride
CDK6: cyclin-dependent kinase 6
CDKN1A: cyclin-dependent kinase inhibitor 1 A
CELF2-AS2: CUGBP Elav-like family member 2 antisense RNA 2
CIB4: calcium and integrin binding family member 4
CL/F: apparent oral clearance
C_max_: maximum concentration
CMC-Na: carboxymethyl cellulose sodium
C_min_: minimum concentration
COX-2: cyclooxygenase-2
DRF: *Dichondra repens* J.R.Forst.and G.Forst.
DDB: dimethyl dicarboxylate biphenyl
D-GlaN: d-galactosamine
DPPH: 1,1-Diphenyl-2-picrylhydrazyl radical 2, 2-Diphenyl-1-(2,4,6-trinitrophenyl) hydrazyl
DTNB: 5,5′-Dithiobis-(2-nitrobenzoic acid)
DNA: deoxyriboNucleic acid
ESI-MS: electrospray ionization mass spectrometry
ELISA: enzyme linked immunosorbent assay
FACS: fluorescent-activated cell sorting
FOS: murine osteosarcoma viral oncogene homolog
FRAP: ferric reducing antioxidant property
5-FU: 5-Fluorouracil
GAP: good agricultural practice
GC: gas chromatograph
GC-MS: gas chromatography-mass spectrometry
GPNMB: glycoprotein (transmembrane) NMB
GPx: glutathione peroxidase
H19: *Homo sapiens* H19 imprinted maternally expressed transcript
HBV: hepatitis B virus
HbsAg: hepatitis B surface antigen
HCC: hepatocellular carcinoma cell
iNOS: inducible nitric oxide
H and E: hematoxylin-eosin staining
HepG2: human liver hepatocellular carcinoma cells
HepG2.2.15: HepG2 cell lines transfected with HBV gene stably
HepG2 A64: both lamivudine- and entecavir-resistant HepG2 cell lines harboring mutants in rtL180 M plus rtM204V plus rtT184 L
HPLC: high performance liquid chromatography
HRESIMS: high resolution electrospray ionization mass spectroscopy
ICR: institute of cancer research
IC_50_: 50% inhibiting concentration
IL-21 R: interleukin 21 receptor
IS: infrared spectrum
KLHDC7B: kelch domain containing 7B
KM: Kunming
LAMβ3: laminin β3
LC-MS/MS: liquid chromatography tandem mass spectrometry
LPS: lipopolysaccharide
MBC: minimum bactericidal concentration
MDA: malondialdehyde
MDR: multi-drug resistance
MIC: minimum inhibitory concentration
MMP: mitochondrial membrane potential
MRPL23-AS1: mitochondrial ribosomal protein L23 antisense RNA 1
MS: mass spectrometer
MTD: maximum tolerated dose
MTS: Matijin-Su
MTT: 3-(4,5-dimethyl-2-thiazolyl)-2,5-diphenyl-2-H-tetrazolium bromide
NDR: n-butanol extracts of DRF
NF-κB: nuclear factor kB
NMR: nuclear magnetic resonance
NO: nitric oxide
NS: normal saline
PCR: quantitative real time polymerase chain reaction
PER: petroleum ether extracts of DRF
PGE2: prostaglandin E2
PKC: protein kinase C
Pyk2: protein tyrosine kinase two
QGY-7701: human hepatocellular carcinoma cell line
RAW 264.7: mouse leukemia cells of monocyte macrophage
ROS: reactive oxygen species
SAR: structure-activity relationship
SD: standard deviation
SI: selective index
SMMC-7721: human hepatocellular carcinoma cell line
SOD: superoxide dismutase
SPF: specific pathogen free
SPINK4: serine peptidase inhibitor Kazal type 4
SYT4: synaptotagmin IV
t_1/2_: half-life
TAA: thioacetamide
TBil: total bilirubin
TC_50_: 50% toxic concentration
TC: total cholesterol
TCM: traditional Chinese medicine
TG: triglyceride
TLC: thin layer chromatography
TNF: tumor necrosis factor
V/F: apparent volume of distribution
ZNF503-AS1: zinc finger protein 503 antisense RNA 1
